# The electroretinogram b-wave amplitude: a differential physiological measure for Attention Deficit Hyperactivity Disorder and Autism Spectrum Disorder

**DOI:** 10.1186/s11689-022-09440-2

**Published:** 2022-05-06

**Authors:** Irene O. Lee, David H. Skuse, Paul A. Constable, Fernando Marmolejo-Ramos, Ludvig R. Olsen, Dorothy A. Thompson

**Affiliations:** 1 Behavioural and Brain Sciences Unit, Population Policy and Practice Programme, UCL Great Ormond Street Institute of Child Health, University College London, London, UK; 2 Caring Futures Institute, College of Nursing and Health Sciences, Flinders University, Adelaide, South Australia Australia; 3 Centre for Change and Complexity in Learning, University of South Australia, Adelaide, Australia; 4 Department of Molecular Medicine (MOMA), Aarhus University, Aarhus, Denmark; 5 The Tony Kriss Visual Electrophysiology Unit, Clinical and Academic Department of Ophthalmology, Sight and Sound Centre, Great Ormond Street Hospital for Children NHS Trust, London, UK; 6 UCL Great Ormond Street Institute of Child Health, University College London, London, UK

**Keywords:** Electroretinogram, Neurotransmission Imbalance, Differentiation, Physiological Marker, Glutamate, GABA, ADHD, ASD

## Abstract

**Background:**

Attention Deficit Hyperactivity Disorder (ADHD) is the most prevalent childhood neurodevelopmental disorder. It shares some genetic risk with Autism Spectrum Disorder (ASD), and the conditions often occur together. Both are potentially associated with abnormal glutamate and GABA neurotransmission, which can be modelled by measuring the synaptic activity in the retina with an electroretinogram (ERG). Reduction of retinal responses in ASD has been reported, but little is known about retinal activity in ADHD. In this study, we compared the light-adapted ERGs of individuals with ADHD, ASD and controls to investigate whether retinal responses differ between these neurodevelopmental conditions.

**Methods:**

Full field light-adapted ERGs were recorded from 15 ADHD, 57 ASD (without ADHD) and 59 control participants, aged from 5.4 to 27.3 years old. A Troland protocol was used with a random series of nine flash strengths from −0.367 to 1.204 log photopic cd.s.m^−2^. The time-to-peak and amplitude of the a- and b-waves and the parameters of the Photopic Negative Response (PhNR) were compared amongst the three groups of participants, using generalised estimating equations.

**Results:**

Statistically significant elevations of the ERG b-wave amplitudes, PhNR responses and faster timings of the b-wave time-to-peak were found in those with ADHD compared with both the control and ASD groups. The greatest elevation in the b-wave amplitudes associated with ADHD were observed at 1.204 log phot cd.s.m^−2^ flash strength (*p* < .0001), at which the b-wave amplitude in ASD was significantly lower than that in the controls. Using this measure, ADHD could be distinguished from ASD with an area under the curve of 0.88.

**Conclusions:**

The ERG b-wave amplitude appears to be a distinctive differential feature for both ADHD and ASD, which produced a reversed pattern of b-wave responses. These findings imply imbalances between glutamate and GABA neurotransmission which primarily regulate the b-wave formation. Abnormalities in the b-wave amplitude could provisionally serve as a biomarker for both neurodevelopmental conditions.

**Supplementary Information:**

The online version contains supplementary material available at 10.1186/s11689-022-09440-2.

## Background

Attention Deficit Hyperactivity Disorder (ADHD) is a neurodevelopmental condition and one of the most common mental disorders in children and adolescents [1], affecting approximately 5% of children worldwide [2–4]. The characteristics of individuals with ADHD include inattention, hyperactivity and impulsivity [4]. Many children with ADHD continue to show symptoms in adolescence and adulthood, frequently struggling with various aspects of their lives [5, 6]. ADHD in adults has historically been underdiagnosed and has received less research attention than childhood ADHD [7]. ADHD is associated with delayed cortical maturation in many regions of the brain, including the visual cortex [8, 9]. ADHD has a strong biological underpinning, including alterations in the dopaminergic neurotransmitter system, leading to neuropsychological deficits [10] and affecting attention, working memory and aspects of visual perception such as colour discrimination, visual search and visual processing speed [10–12], along with the potential impact of these visual problems on education in children and on working or driving performances in adults [13].

Autism Spectrum Disorder (ASD) is also a neurodevelopmental disorder characterised by persistent deficits in co-occurring impairments of social reciprocity and social communication, repetitive patterns of behaviour and atypical responses to sensory input or unusual interests in aspects of the environment [4, 14]. Both ADHD and ASD exhibit some genetic and behavioural overlap and have abnormalities in similar brain systems, in particular the frontal and cerebellar regions [15]. Both disorders are highly heritable and share high comorbidity [16, 17]. Between 20–50% of children with ADHD have ASD traits and 30–80% of ASD children have co-occurring ADHD [17–19]. For years, ADHD-related deficits in ASD were considered a phenocopy, which prevented their formal co-diagnosis in both DSM-IV and ICD-10 [15, 20]. This diagnostic rule is no longer applied in DSM-5 or ICD-11 [4, 15]. Disorder-specific physiological biomarkers that differentiate these two conditions could further our understanding of their underlying neurobiology and their relative contribution to an individual’s phenotype.

**Fig. 1:**
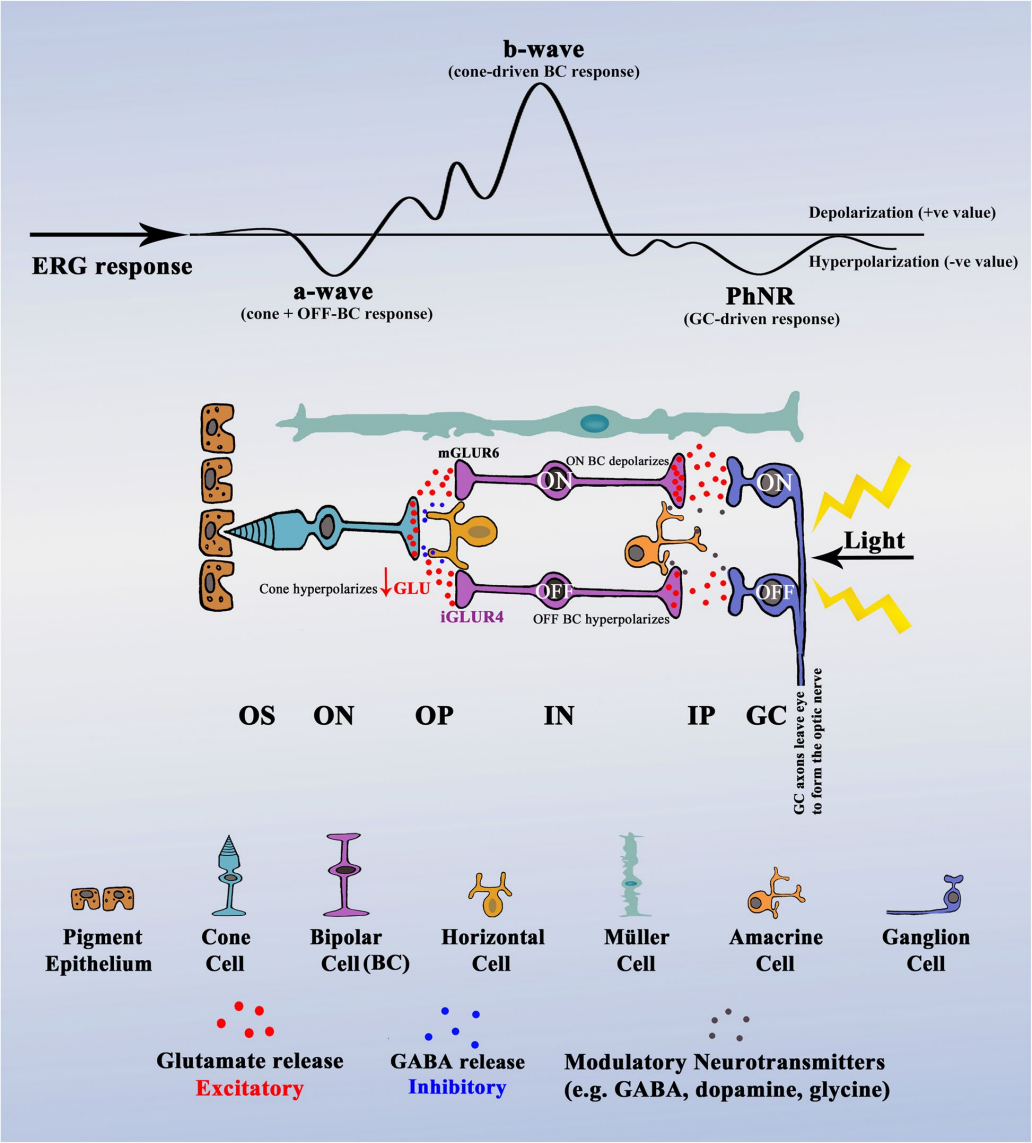
Schematic diagram of the retinal pathway generating the electroretinogram in response to light. The schematic diagram displays the cellular component of the retina and the retinal pathway in response to light generating the electroretinogram (ERG) waveform. Light passes through the transparent retinal layers before reaching the photoreceptor chromophores which absorb the photons. The cone photoreceptor outer segment subsequently hyperpolarises, shutting off glutamate release into the post photoreceptor synapse. This hyperpolarisation is recorded as the a-wave in the electroretinogram waveform. Glutamate has opposite effects on the ON- and OFF-bipolar cells. Decreased glutamate binding on the mGLUR6 receptor starts a cascade of signals that open the transient receptor potential cation channel, subfamily M, member 1 (TRPM1) channel which depolarises the ON-bipolar cells and increases glutamate release to the ON-ganglion cell [31]. In contrast, the OFF-bipolar cell becomes hyperpolarised by the reduction of glutamate release from the cone cell binding on the iGLUR4 receptor, resulting in decreased glutamate release toward the OFF-ganglion cell. The b-wave amplitude is the summation of ON- and OFF-bipolar cell responses. The Photopic Negative Response (PhNR) is the summation of ON- and OFF-ganglion cell responses and contributions of Müller cell potassium currents. Glu, glutamate release; red arrow pointing down means reduced; mGLUR6, metabotropic glutamate receptor 6; iGLUR4, ionotropic glutamate receptor 4. Retinal layers: OS, Outer Segment; ON, Outer Nuclear; OP, Outer Plexiform; IN, Inner Nuclear; IP, Inner Plexiform; GC, Ganglion Cell

The eye and neural retina provide a window to the neurobiology of the brain and have been of growing interest to those studying neurodegenerative and psychiatric disorders [21–27]. The retina has three highly organised cellular layers that are interconnected by two synaptic layers (see Fig. 1). Normal retinal neural function depends, like elsewhere in the brain, upon the balance of GABAergic (inhibitory) and glutamatergic (excitatory) neurotransmission. The synaptic networks in the retina change according to the strength of the background or flashed light. Under light-adapted (LA) conditions, the retinal networks are driven by the hyperpolarisation of light-activated cone photoreceptors, which synapse with a triad of cells: ON- and OFF-bipolar cells and horizontal cells [28]. Cone cell hyperpolarisation reduces the glutamate released to the ON- and OFF-bipolar cells. These bipolar cells contain different types of glutamate receptors which produce opposite responses to glutamate. ON-bipolar cells use slower metabotropic (ligand sensitive) glutamate receptors (primarily mGLUR6) that invert the cone hyperpolarisation into depolarisation. In contrast, OFF-bipolar cells use fast ionotropic glutamate receptors (iGLUR4, ligand-gated cation channels of the AMPA/Kainate class) that conserve hyperpolarisation [29]. Glutamate thus regulates excitatory signalling between the cones and bipolar cells. GABA, the most abundant inhibitory neurotransmitter in the brain, is presumed to allow the horizontal cells in the retina to modulate the balance and timing and gain of the cone signalling pathways [30].

**Fig. 2:**
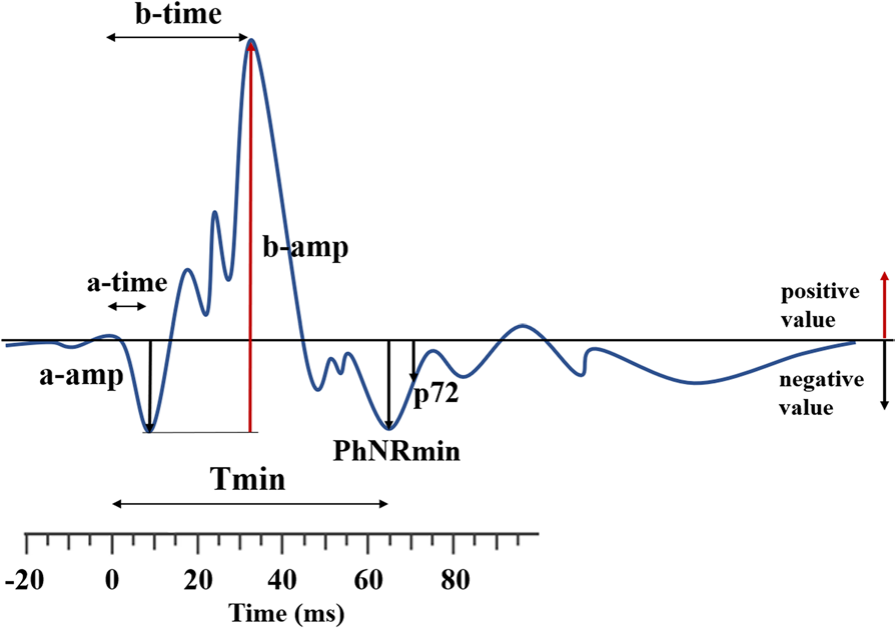
Different parameters of the ERG waveform. The ERG response starts at time 0 followed by the a-wave, b-wave, and Photopic Negative Response (PhNR). Description of the ERG waveform parameters: a-amp, a-wave amplitude, an amplitude from the baseline to the a-wave trough; a-time, a-wave time-to-peak, from the light onset to a-wave trough; b-amp, b-wave amplitude, measured from a-wave trough to b-wave peak; b-time, b-wave time-to-peak, from the light onset to the time when the b-wave amplitude peaks; p72, PhNR amplitude from baseline to the waveform at 72 ms post-stimulus onset; PhNRmin, PhNR amplitude measured as the most negative point from the baseline within the time window of 55 and 95 ms following stimulus onset; Tmin, time of PhNR at a minimal amplitude occurred within the 55–95-ms window

Light entering the eye causes retinal cell membranes to hyperpolarise and depolarise at different times. The net signal can be recorded at the front of the eye as the electroretinogram (ERG) waveform. This can be observed as a voltage change over time following the onset of a light flash (see Fig. 2). The ERG waveform has three features, each of which has distinct cellular origins. The first negative trough is called the a-wave and reflects the rapid hyperpolarisation of cone photoreceptors and the associated OFF-bipolar cells. It is followed by the b-wave, a positive peak which reflects the slower depolarisation of the ON-bipolar cells and the recovery repolarisation of the hyperpolarised OFF-bipolar cells. The third feature is the trough after the b-wave termed the photopic negative response (PhNR) which is associated with retinal ganglion cell activity [32–34], mediated by potassium currents in the Müller glia cells which extend through the retina [35]. Changes in the size and timing of the peaks and troughs of the ERG reflect the proportionate contributions of specific retinal cells in signalling the onset and offset of a flash of light and the balance of glutamate and GABA neurotransmission responding to that stimulus.

The ERG is increasingly being used to distinguish between psychiatric conditions, such as schizophrenia from bipolar disorder, and to investigate the effects of drug treatment in conditions such as depression [36–41]. In children and adults with ASD, reduced ERG b-wave amplitudes, compared to those of typical controls, have been observed under both dark-adapted (DA) and LA conditions and are considered to reflect an imbalance in glutamate and GABA signalling [42–45]. In ADHD, not much is known about the retina’s response to light, though increased background retinal noise was reported recently [1, 46]. The origin of the background retinal noise in ADHD is unexplained, but it correlates with measures of inattention symptoms [1, 47]. Reports show an association of the genetic variants involved in glutamate neurotransmission with the severity of hyperactivity and impulsivity, implying a role for ionotropic and metabolic glutamate receptors in the pathogenesis of ADHD [48, 49].

In this study, we investigated whether the LA-ERG waveform is different in ADHD individuals compared to ASD and control individuals.

## Methods

### Participants

Fifteen individuals with ADHD (age mean ± SD, 15.3 ± 3.5 years) and fifty-seven participants with ASD (13.7 ± 4.8 years) were recruited. Clinical evaluations were made by specialist paediatric psychiatrists and clinical psychologists in clinics for children with neurodevelopmental disorders at London’s Great Ormond Street Hospital for Children and at local clinics in the UK and South Australia. Diagnostic assessments were supported by parental interviews (the Developmental, Dimensional and Diagnostic Interview, 3Di) [50], school reports and structured observations (Autism Diagnostic Observation Schedule (ADOS)). In this study, we selected children who, after comprehensive clinical evaluation, were considered to meet the diagnostic criteria (DSM-IV, DSM-5 or ICD-10), for ASD without co-occurring ADHD or ADHD without significant ASD traits. Other exclusion criteria included a family history of ocular disease or strabismus, any history of brain trauma or pathology, a history of epileptic seizures in the last year, full-scale IQ < 65 or an ability to follow simple verbal instructions.

Male participants predominated in both groups: ADHD (53%) and ASD (75%). All had normal range intelligence: mean full-scale IQ 92.9 ± 14.2 in ADHD and 100.5 ± 19.4 in ASD. ADHD severity scores were based on the ICD-10 Research Diagnostic Criteria and calculated from measures of hyperactivity, impulsivity and inattention provided by parents/carers and schoolteachers for both ADHD and ASD participants. ADHD severity scores range from 0 (no symptoms) to 6; the mean ADHD severity scores in the ADHD and ASD groups were 3.9 ± 0.8 and 2.0 ± 0.7, respectively (Table 1). The ADOS total scores were used for the analysis, and the ASD severity score was a standardised score calculated from the ADOS total score according to the methods of Gotham et al. [51]. The mean of the ADOS total scores and ASD severity score of the ASD group was 10.9 ± 4.7 and 6.2 ± 2.0, respectively. No ADOS scores were obtained from the ADHD cohort.

**Table 1:** Participant demographic information

	CTL	ADHD	ASD (without ADHD)
***N***	59	15	57
**Male (%)**	53%	53%	75%
**Age (years)**			
Mean (M)	13.3 ± 4.6	15.3 ± 3.5	13.7 ± 4.8
Median (Mdn)	12.8	15.5	13.3
Range	5–25	8–20	6–27
**Iris colour index**^**a**^	1.25 ± 0.13	1.26 ± 0.12	1.20 ± 0.11
**Vert**	2.4 ± 0.8	1.5 ± 1.3	2.2 ± 0.8
**Ethnicity (%)**			
Caucasian	35 (59)	11 (73)	47 (82)
Asian	18 (31)	2 (13)	3 (5)
Afro-Caribbean	0	1 (7)	1 (2)
Latino	0	1 (7)	1 (2)
Mixed	6 (10)	0	5 (9)
**FSIQ (*n*)**	–	92.9 ± 14.2 (10)	99.6 ± 18.9 (37)
**ADHD severity**^**b**^**(*n*)**	–	4.0 ± 0.9 (11)	2.0 ± 0.8 (25)
Hyperactivity		5.5 ± 2.3	2.0 ± 1.8
Impulsivity		4.1 ± 1.9	3.1 ± 1.9
Inattention		9.5 ± 3.4	6.3 ± 4.0
**ADOS total**	–	–	11.0 ± 4.8 (34)
**ASD severity**	–	–	6.2 ± 2.0 (34)
**NMed**	–	2^c^	8

All data are presented as M ± SD

*CTL* Control, with no ASD or ADHD proband in their first-degree family; *N* Number of participants; *FSIQ* Full Scale IQ; *n* Number of data collected, as some participants diagnosed in the local clinics did not have their phenotypic data; *ADOS total* Autism Diagnostic Observation Schedule total score (0–28, higher the score more severe); *ASD severity* Autism severity score (range 1–10, ≥ 4 is ASD, higher the score more severe); *NMed* Number of participants taking medications before testing

^a^The distribution of the iris colour index of each group is displayed in Additional file 1: Fig. S1; *Vert* Vertical distance (mm) of the electrode below the lower eyelid

^b^*ADHD severity* ADHD severity score (maximum is 6, higher more severe), each ADHD subset score (hyperactivity, impulsivity and inattention) was the combined score evaluated by parents/carers and schoolteachers

^c^Another five ADHD subjects had been tested before and after taking ADHD medications

The comorbidities and medications of participants in both cohorts are listed in Additional file 1: Table S1A. Five ADHD participants were tested before and after taking their prescribed methylphenidate medications (see Additional file 1: Table S1B). At the time of testing after taking their medication, the methylphenidate levels in their blood had reached over 80% of maximum and were within the duration of drug action [52–54].

Fifty-nine typically developing controls were recruited with no familial history of ASD or ADHD and no diagnosed mental health condition. The control group’s mean age was 13.3 ± 4.6 years. One child had been diagnosed with diabetes. No control participants were taking psychoactive medications, and all had normal or corrected-to-normal visual acuity. Written informed consent was obtained from the parent, guardian or the participant (if older than 16 years of age) in all three groups, and the study was reviewed by the appropriate Institutional Ethics Committees.

### Electroretinogram

The ERG is a clinical test defined by the International Society for Clinical Electrophysiology of Vision (ISCEV) standard [55]. A customised LA full-field ERG series was performed using white light at nine flash strengths (see all the variables in Table 2) that were presented in random order at 2 Hz, on a 40-cd.m^−2^ white background with an average of 60 trials to obtain the waveform with repeated recordings for both eyes. The nine randomised flash strengths were then followed by the ISCEV standard flash 3.0 cd.s.m^−2^ on a 30-cd.m^−2^ white background presented at 2 Hz with 30 samples averaged, to generate the waveform. This custom protocol was programmed in the RETeval (LKC Technologies Inc., Gaithersburg, MD, USA), which was used for all recordings, using a self-adhesive skin electrode positioned 2–3 mm below the participant’s lower eyelid in accordance with the manufacturer’s recommendation. The RETeval automatically stopped recording if pupil tracking was lost (due to poor fixation, pupil size < 1.8mm or the electrode impedance was > 5 kΩ. For further details, see [43, 56].

**Table 2:** The description of all the variables in the GEE model

**Independent Variable (IV) **(categorical in italic)		**Dependent Variable (DV)**	
**A**	**A**ge	**a-amp**	a-wave amplitude (µV)
**I**	**I**ris colour index (iris pigmentation measure)	**a-time**	a-wave time-to-peak (ms)
***V***	**V**ertical location of the electrode (five levels, mm)	**b-amp**	b-wave amplitude (µV)
***G***	Participant **G**roup (ASD, control and ADHD)	**b-time**	b-wave time-to-peak (ms)
***e***	Left **e**ye or right eye	**p72**	PhNR amplitude at time 72ms (µV)
***s***	**s**ex (male or female)	**PhNRmin**	PhNRmin amplitude (µV)
***M***	**M**edication (taken or not-taken before the ERG recording)	**Tmin**	Tmin (ms)
***E***	**E**thnicity	**p-ratio**	p72/(b-wave amplitude – a-wave amplitude)
***FS***	**F**lash **S**trengths at: -0.367, -0.119, 0.114, 0.398, 0.602, 0.799, 0.949, 1.114 and 1.204 log phot cd.s.m^-2^ & ISCEV LA3 standard flash (0.477)	**w-ratio**	PhNRmin/(b-wave amplitude – a-wave amplitude)
***F*S***•G*	‘•’ stands for interaction.		All DVs are numeric, see description in Fig. 1.

The model examined via GEE was *FS *+ *V *+ *G *+ *E *+ I + s + *M *+ A + *e *+ *FS•G*

### Statistical analysis

Data were analysed via generalised estimating equations (GEEs). GEEs are non-likelihood models akin to linear mixed-effects models (LMM) in that they deal with clustered data in cases of repeated measures [57]. GEEs allow defining the family of the response distribution in shapes other than the normal distribution, which is the only option in LMMs [58, 59]. Akin to the goodness-of-fit (GoF) metric Akaike Information Criterion, GEE models’ GoF was assessed via quasi-information criterion (QIC) which is a quasi-likelihood metric under the independence model information criterion [60].

In all models, ‘participants’ as random effects were entered as the vector which identifies clusters, whilst the covariates listed in Table 2 were entered as fixed effects. Models in which the interaction *FS•G* were significant were further assessed via QIC by retaining significant variables signalled in the full model but leaving out ‘ethnicity’ (see Additional file 1: Table S2). Such examination consisted of 10 repetitions of 10-fold cross-validation was assessed via a Gaussian cross-validation metrics. Non-parametric multiple pairwise comparisons [61] were performed to identify significances amongst the three groups at each flash strength. The aim was to ascertain the combination of flash strength and dependent variable that showed the best potential to differentiate between the groups. The effect of ADHD medications on the ERG measures was analysed to determine if taking medicine before or after (*M*_*ba*_) influenced any of the dependent variables. This analysis was performed via robust linear models. The resulting models were examined via type III ANOVA. If a significant interaction was not present, then the main effects were not analysed further.

The change of b-wave amplitude with flash strength was plotted as the photopic hill and analysed using a mathematical modelling of combinations of a Gaussian function that represents the OFF-pathway and a logistic function that represents ON-pathway contributions according to its parameter maximal Gaussian amplitude (*G*_*b*_) and maximal saturated amplitude (*V*_*b*max_), respectively [62]. As background luminance increases, both components shift to the right on the luminance axis. The Gaussian component increases in amplitude as the logistic growth function component decreased in amplitude. The photopic hill equation model is formulated as follows.

$$y={G}_b\left[{\left(\frac{I}{\mu}\right)}^{\frac{\ln \left(\frac{\mu }{I}\right)}{B^2}}\right]+\frac{V_{b\max }I}{I+{\sigma}_b}$$

All the five parameter values, including the measures of the width of the Gaussian curve (*B*^2^), flash strength (*I*), semi-saturation flash strength (*σ*), and *G*_*b*_ and *V*_*b*max_ of each group were generated using non-linear curve-fitting in OriginPro 2019 following the photopic hill equation which was inputted as a customised equation in the Fitting Function Builder. The parameters between the groups were compared by one-way ANOVA. The correlation plot network between the ERG measures and the cohort phenotypes was produced in Origin 2021b.

A *p-*value < .005 was adopted in all the analyses in this study as a cut-off of statistical significance [63]. All R codes for the analysis, datasets and outputs of this study are available at the FigShare repository (https://figshare.com/s/5176e951c419612e6273).

## Results

GEE was performed in this analysis to compare all the ERG parameters in repeated measures amongst the ADHD, control and ASD groups. Additional file 1: Table S2 summarises the effects of each independent variable on the ERG parameters. Further analysis based on the quasi-likelihood theory to examine the interaction (*FS•G*) of flash strength and the groups demonstrated a statistically significant differences on three variables (see QIC_2_ in Additional file 1: Table S2). Three ERG variables significantly differentiated the groups (*FS•G*, df = 18) that these were b-wave amplitude (*p* = 7.8 × 10^−7^), b-wave time (*p* = 1.4 × 10^−8^) and the p72 amplitude (*p* = .001).

**Fig. 3:**
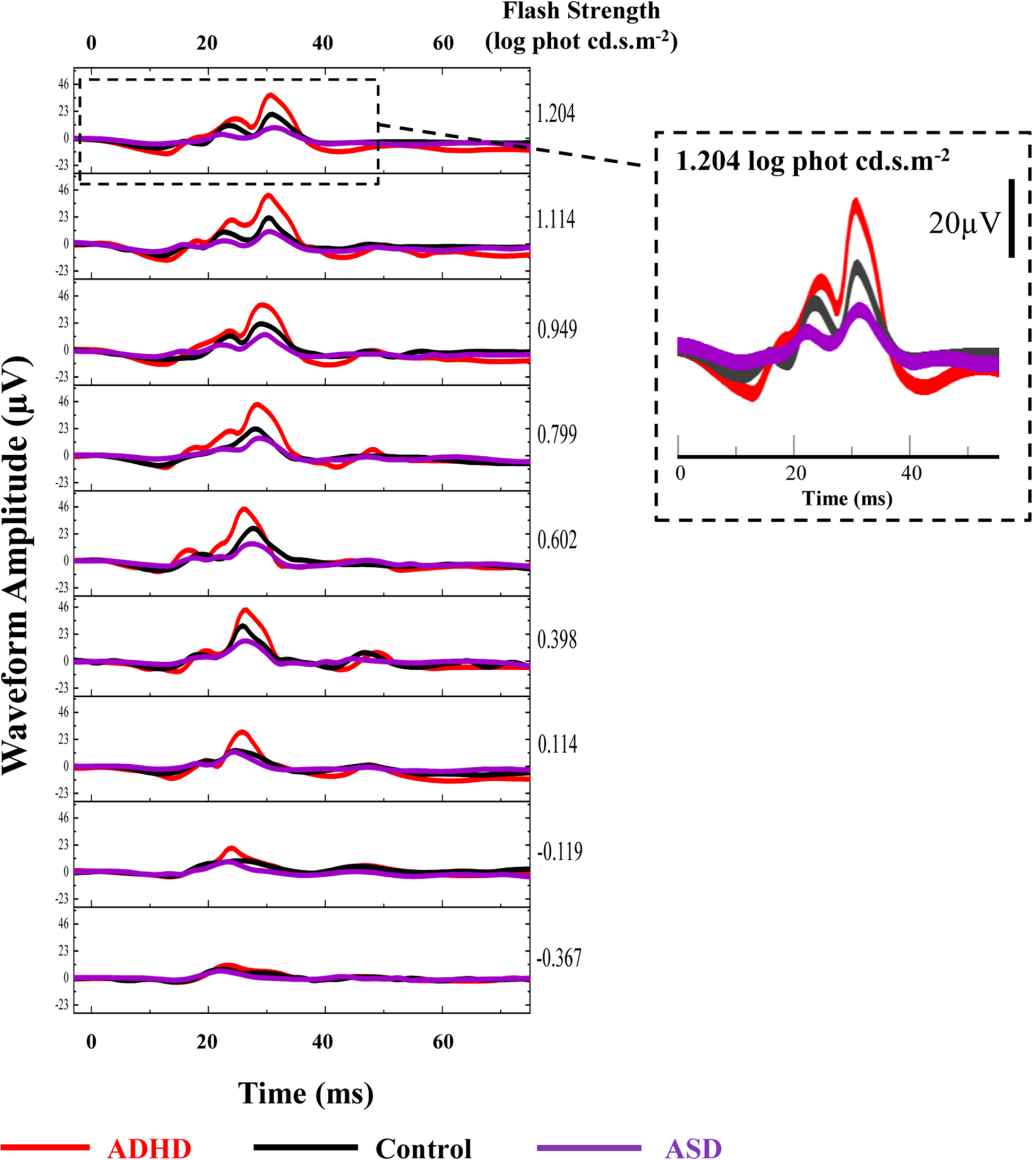
Comparison of the representative light-adapted ERG waveforms of one individual from each group. The ERG waveforms of each ADHD, control and ASD individual at the nine randomised flash strengths are displayed on the left panel. The right panel enlarges the image of the representative ERG waveforms produced by the 1.204 log phot cd.s.m^−2^ flash strength, which showed the most significant differences amongst these individuals. The b-wave amplitudes of ADHD are all distinctively higher than both ASD and control individuals at all light strengths. The b-wave amplitudes of ASD are lower than the controls from 0.398 to 1.204 log phot cd.s.m^–^^2^

The finding showed a noticeable elevation of the b-wave amplitude in the ADHD group compared to the control and ASD groups, whereas the ASD b-wave amplitude was reduced compared to comparison control. Figure 3 shows the ERG waveforms for a representative participant from each group at the nine randomised flash strengths.

### b-wave amplitude—ON- and OFF-bipolar cells

**Fig. 4:**
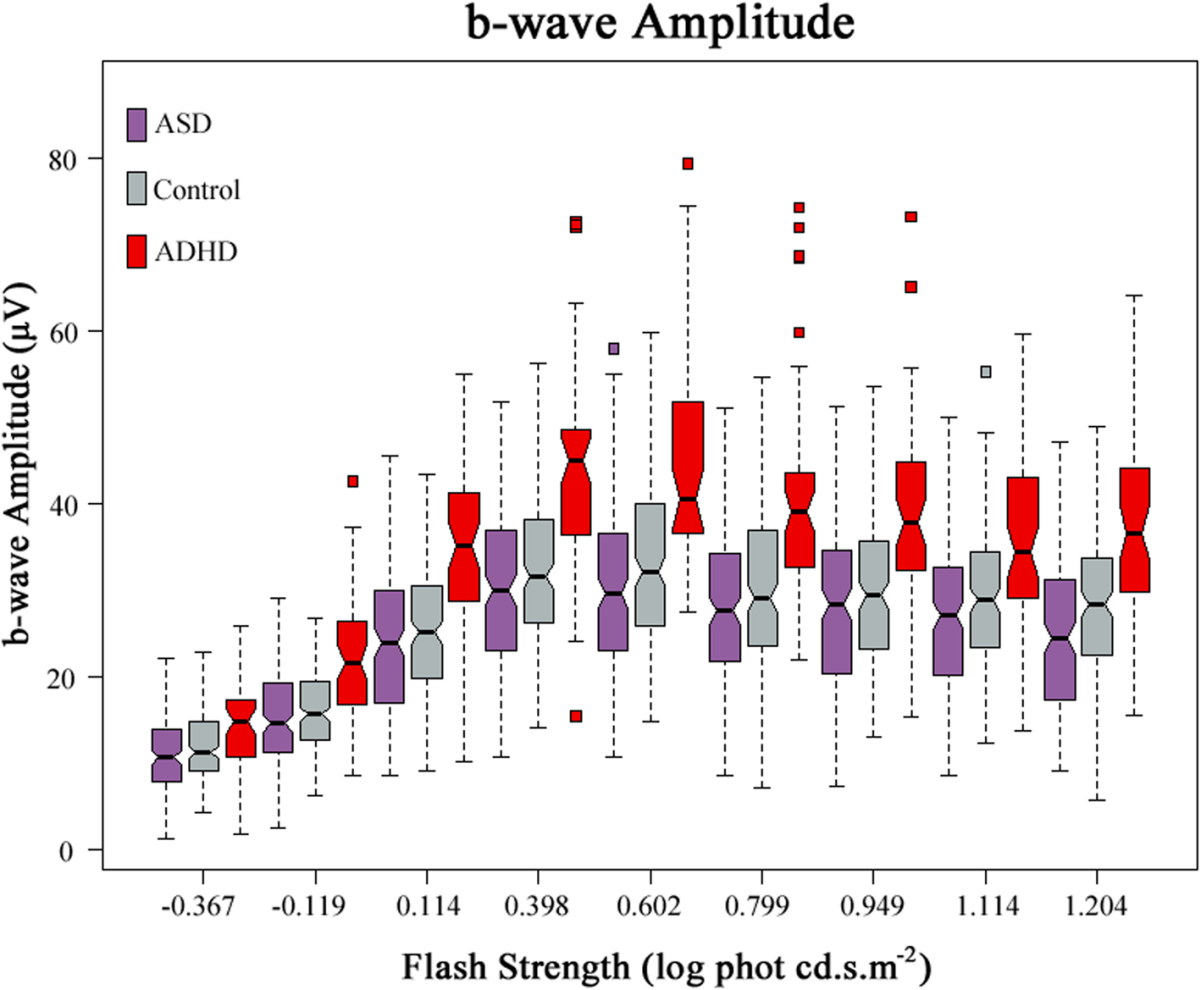
Comparison of the b-wave amplitudes of the ADHD, Control and ASD groups. For each flash strength, a set of three boxplots representing ASD, Control and ADHD (from left to right) are displayed. The medians and 95% CIs of each group are presented. The medians of ADHD are distinctively higher than those of the control and ASD groups at all flash strengths

**Fig. 5:**
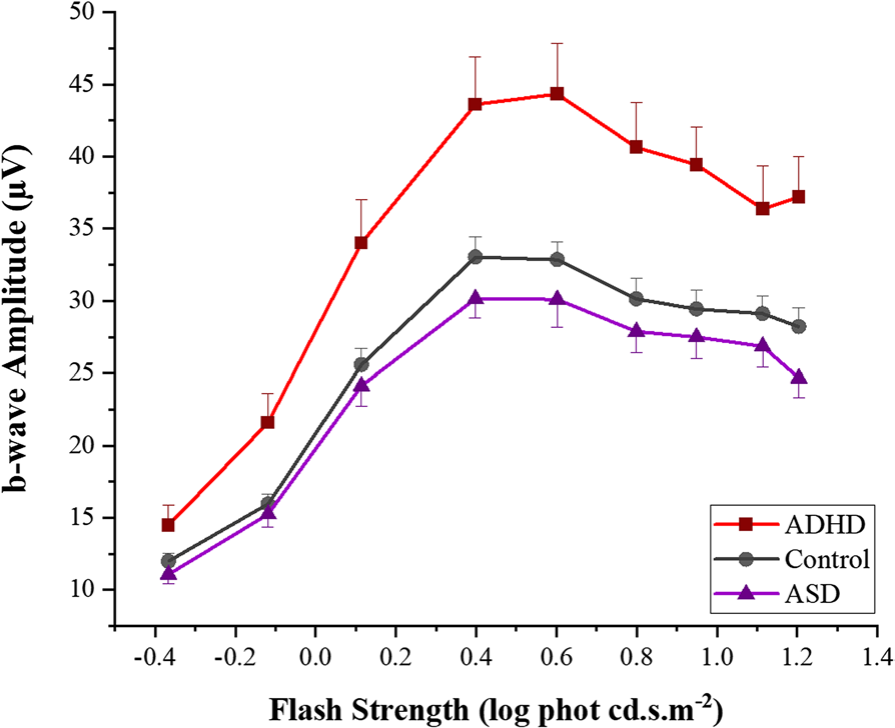
The photopic hill of the b-wave amplitudes of the ADHD, Control and ASD groups. The mean b-wave amplitudes with 95% CIs for each group are plotted at the nine flash strengths

Figure 4 displays the medians and 95% CIs of the b-wave amplitudes of the three groups. The median b-wave amplitudes of the ADHD group are significantly higher than those of the control individuals and ASD probands at all flash strengths (all *p-*values < .005). The photopic hill in Fig. 5 describes the change of the b-wave amplitude against each flash strength. The modelling revealed a significant difference in the two main parameters, maximal saturated amplitude (*V*_*b*max_, *p* < .00001) and maximal Gaussian amplitude (*G*_*b*_, *p =* .0038) in ADHD compared to the other groups (see Table 3). The *p*-values of pairwise comparisons between the groups are listed in Additional file 1: Table S3. Two flash strengths, 0.398 and 1.204 log cd.s.m^−2^, demonstrated all statistically significant differences between the groups. The b-wave amplitudes of ADHD, control and ASD at 1.204 log cd.s.m^−2^ were 37.2 ± 10.3 μV, 28.2 ± 8.6 μV and 24.7 ± 8.9μV, respectively (*p* = 3.98 × 10^−12^) (see Additional file 1: Table S4A).

**Table 3:** Comparison of the photopic hill parameters

	ADHD	Control	ASD	***F***	Overall ***p***-value
***G***_***b***_	14.99 ± 1.2	10.08 ± 1.4	9.33 ± 1.3	5.57	.0038*
***B***^**2**^	1.04 ± 0.2	.65 ± 0.2	.93 ± 0.3	.71	.49
***V***_***b*max**_	38.18 ± 1.3	30.24 ± 1.1	26.61 ± 1.4	21.61	< .00001*
***σ***	.78 ± .08	.70 ± .07	.66 ± .09	.58	.56
***μ***	2.58 ± .2	2.49 ± .2	2.59 ± .3	.054	.95
**log (*μ*)**	.412	.396	.413		

*G*_*b*_ Maximal Gaussian amplitude, *B*^*2*^ Measure of the width of the Gaussian curve, *V*_*bmax*_ Maximal saturated amplitude, *σ* Semi-saturation flash strength that evokes a half-maximal response of the b-wave amplitude, *μ* Peak flash strength (phot cd.s.m^−2^). *p < .005

### b-wave time-to-peak

**Fig. 6:**
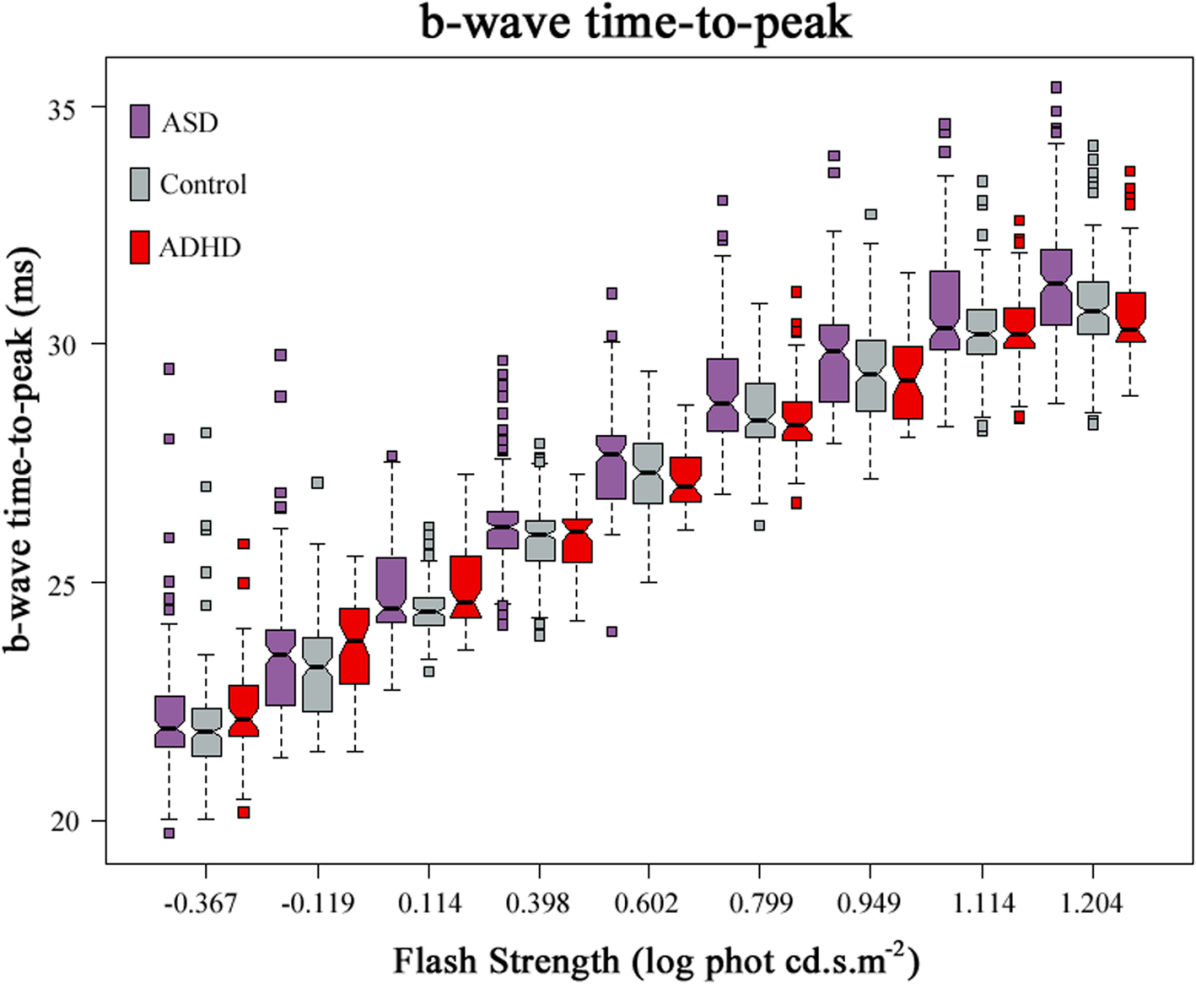
Boxplots showing the comparisons of the b-wave time-to-peak of the ADHD, ASD and Control groups. For each flash strength, a set of three boxplots representing ASD, Control and ADHD (from left to right) are displayed. The medians and 95% CIs of each group are presented

In all groups, the b-wave time-to-peak gets later as the flash strength increases. The b-wave time-to-peak differed only at some flash strengths by pairwise comparisons amongst the groups (see Additional file 1: Table S3). The main finding was that the higher flash strength of 1.204 log phot cd.s.m^−2^ produced a faster b-wave time-to-peak for ADHD compared to the ASD groups as the flash strength increased, from 0.398 to 1.204 log cd.s.m^−2^. The b-wave time-to-peak of ASD is also slower than the control group at the high flash strength of 1.204 log phot cd.s.m^−2^. However, the differences in the b-wave time-to-peak between the groups were similar at low flash strengths (see Fig. 6 and Additional file 1: Table S4B). The most significant difference in b-time-to-peak was at 1.204 log phot cd.s.m^−2^ amongst the three groups (*p* = .001), which the b-time-to-peak of ADHD was 30.6 ± 1.1 ms, compared to 30.8 ± 1.1 ms in Control and 31.2 ± 1.8 ms in the ASD group.

### The Photopic Negative Response p72 amplitude—Retinal ganglion cells and Müller cells

**Fig. 7:**
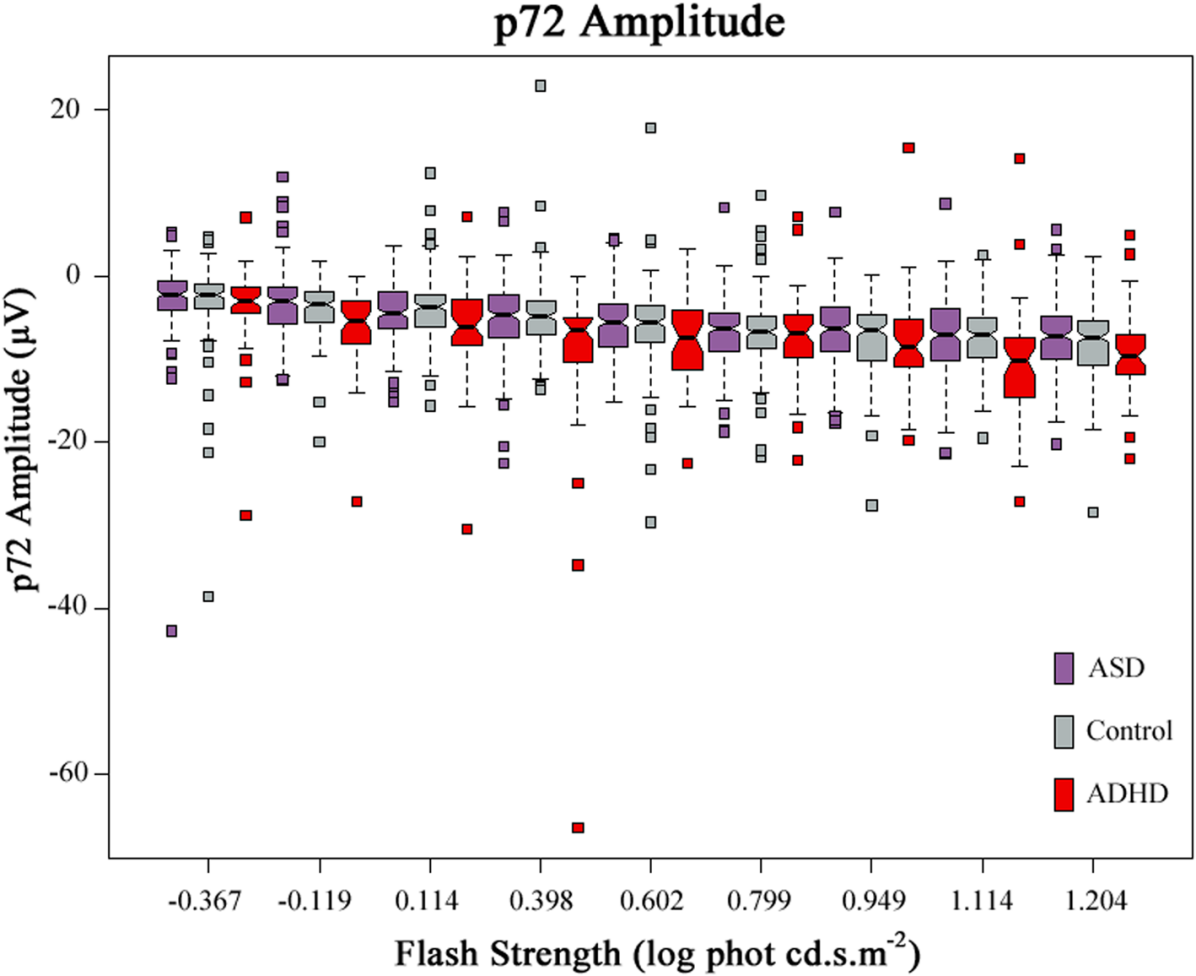
The Photopic Negative Responses (PhNR) at *t* = 72 ms (p72). The amplitudes of PhNR, p72, of ADHD, Control and ASD groups are shown at all flash strengths. For each flash strength, a set of three boxplots representing ASD, Control and ADHD (from left to right) are displayed. The medians and 95% CIs of each group are presented

Variable differences between the ADHD group and the other groups were found in the PhNR p72 amplitude. Figure 7 shows that the median p72 amplitudes of ADHD were consistently higher than those of the Control and ASD groups at each flash strength (see Additional file 1: Table S4C). The overall significant increases of p72 amplitudes were observed at flash strengths − 0.119 (*p* = 6.04 × 10^−5^), 0.398 (*p* = .0011), 0.477 (*p* < .001), 1.114 (*p* = 6.21 × 10^−5^) and 1.204 log cd.s.m^−2^ (*p* = .003) amongst the groups. The p72 amplitudes of ADHD subjects were significantly higher than the other two groups at flash strengths − 0.119 and 1.114 log phot cd.s.m^−2^. The p72 amplitudes of ADHD, Control and ASD at 1.114 log phot cd.s.m^−2^ were − 10.6 ± 6.3 μV, − 7.3 ± 3.6 μV and − 7.5 ± 4.5 μV, respectively. However, the PhNR parameters did not distinguish ASD from the control group with no significant differences between these groups (*p* > .24).

For the other ERG measures, no significant differences were observed in each parameter between the ADHD and the other groups in the multiple comparisons, including a-wave time-to-peak (*p* = .07), a-wave amplitude (*p* = .17), Tmin (*p* = .03), PhNRmin (*p* = .01), p-ratio (*p* = .01) and w-ratio (*p* = .02). Additional file 1: Fig. S2 shows the ERG responses of these parameters to all flash strengths in each group.

Direct measurements of the ERGs before and after taking the medication methylphenidate in five ADHD participants did not reveal significant interactions between flash strength and ADHD medication nor other parameters (See *FS•M*_*ba*_ in Additional file 1: Table S5, all the *p-*values are between .011 and .96). Additional file 1: Fig. S3 shows no significant effect of ADHD medications on all the ERG measures.

In addition, no significant correlations between the ADHD and ASD phenotypes or full-scale IQ scores were associated with any of the ERG measures (See Additional file 1: Table S6). Additional file 1: Fig. S4 shows the correlation plot network of all the ERG measures, ASD and ADHD phenotypic variables. The b-wave and p72 amplitudes are directly related to ADHD severity and clusters with ADHD phenotypes.

### Specificity and sensitivity of the b-wave amplitude

The most significant differences between the groups for the b-wave amplitude were at the two flash strengths of 0.398 and 1.204 log phot cd.s.m^−2^ (see the peak and the plateau of the photopic hill in Fig. 5). The b-wave amplitude distinguished the ADHD group from the combined control and ASD group with an area under the receiver operating characteristic curve (AUC ROC) of 0.84 both at 0.398 or 1.204 log phot cd.s.m^−2^ strengths (see Additional file 1: Fig. S5 and Table S7). The b-wave amplitudes of 36.4 μV and 30.5 μV at 0.398 and 1.204 log phot cd.s.m^−2^, respectively, are the predicted b-wave amplitude values for ADHD from the combined ASD and control groups, with 80% sensitivity and 71% at 0.398 log phot cd.s.m^−2^ and sensitivity of 81% and specificity of 70% at 1.204 log phot cd.s.m^−2^. The AUC for the discrimination of ADHD from ASD was better with the AUC ROC of 0.86 and 0.88 at 0.398 and 1.204 log phot cd.s.m^−2^, respectively. The b-wave amplitude cut-off points remained the same, with 80% sensitivity and 67% specificity at 0.398 log phot cd.s.m^−2^, with 84% sensitivity and 57% specificity at 1.204 log phot cd.s.m^−2^.

## Discussion

This is the first report indicating there are significantly larger LA-ERG b-wave amplitudes in children with ADHD compared to those with ASD or typical development. Whilst participants with ADHD had greater than normal responses, those with ASD had smaller than typical b-wave amplitudes. Thus, the ASD and ADHD phenotypes have opposite ERG b-wave amplitude characteristics compared to controls. Although b-time-to-peak and PhNR p72 amplitudes also differentiated ADHD from the ASD and control groups with statistical significance, the b-wave amplitude provided the greatest discrimination, at two flash strengths. These flash strengths represent the peak and plateau of the photopic hill and are shaped by glutamate driven OFF- and ON-bipolar pathways, respectively [62].

There have been previous reports of a reduced ERG b-wave in ASD which have been considered to be associated with altered kinetics and/or different expression levels of the glutamate receptors and transporters at the cone-bipolar-horizontal cell synapse [42, 43]. An ASD mouse model shows a significant structural reduction of photoreceptor and bipolar cell markers, as well as functional defects of the significantly reduced a-wave and b-wave amplitudes compared to controls [64]. The change in the timings of the summation of excitatory and inhibitory signals and hence reduction of the b-wave amplitudes in ASD are linked to an imbalance of glutamate and GABA activities [28, 49, 65]. Research studies have investigated whether there are reduced GABA-A receptors in the ASD brain using both human and mouse models [66]. There were no differences in the GABA-A receptors in any brain region of adults with ASD as well as in any of the ASD mouse models, although adults with ASD displayed alterations in performance during a GABA-sensitive perceptual task [66]. The results suggested that GABA-A receptor availability could be normal in ASD despite the functionally impaired GABA signalling [66]. Post-mortem studies in ASD report abnormalities in the expression of glutamate transporters, GABA-A and GABA-B receptors [67, 68]. Another study has shown an imbalance of excitatory/inhibitory gene expression in ASD but demonstrated that reduced expression of inhibitory genes was more pronounced than those genes related to excitation. It was suggested that the imbalance in ASD was mainly due to GABA disturbances [49, 69].

Variants in the GABAergic gene sets have been reported to be more associated with ASD than ADHD, whereas glutamate gene set activity showed association with ADHD hyperactivity-impulsivity symptoms [49]. A genome-wide copy number variation (CNV) study has identified enrichment of CNVs in metabotropic glutamate receptor gene networks that were overrepresented in cases with ADHD compared to controls, and the findings were replicated in multiple ADHD cohorts [70]. The levels of serum glutamate were two times higher and that of GABA were lower in children with ADHD [71, 72]. Additionally, a research study showed an association of GABA with response inhibition [73]. Many studies using magnetic resonance spectroscopy have reported abnormal levels of glutamate and glutamate/glutamine (Glx) in ADHD adults compared to controls—such as increased glutamate in the cerebellum [74], higher level of striatal glutamate and Glx concentration [49, 75], reduced Glx in the caudate/putamen [7] and reduced glutamate/creatine in both the medial post-frontal cortex and the anterior cingulate cortex [76, 77]. These findings suggest a role for glutamate concentration in fronto-striatal neural functioning during cognitive control [78]. Although a recent study has shown no differences in glutamate and GABA concentration in the anterior cingulate cortex between children with ADHD and controls, glutamate or GABAergic differences in the subregions have not been ruled out [79]. All these findings indicate GABAergic and glutamatergic dysregulation in ADHD. Interestingly, increased glutamate release has been reported from the prefrontal cortex of the spontaneously hyperactive rat [80, 81]. These observations indicate an imbalance of glutamatergic and GABAergic neurotransmission in both ADHD and ASD, and there seems to be greater glutamatergic dysregulation in ADHD and more GABA disturbances in ASD. The nature of that imbalance differentiates these conditions by the alterations of the b-wave formation in reverse direction as demonstrated in this study.

Larger b-wave amplitudes in ADHD could be explained by a lower signal threshold in response to a light flash, and the observation is compatible with other findings of an increase in retinal neuronal noise or non-stimulus driven neuronal activity in ADHD [1, 46]. Elevated neural noise can enhance cell threshold systems by allowing a signal to reach the threshold at intermediate noise intensities through stochastic resonance or facilitation [82]. This typically enhances low signals, which are produced in the retina to dim flash strengths. In ADHD, it is plausible that an abnormal interplay between response mechanisms contributes to the stochastic resonance that extends across the entire signalling range of the retinal cone networks [83]. The timing of the ionotropic synapse (OFF-response) can be changed by glutamate receptor auxillary subunits [84, 85]. The photopic hill model in Fig. 5 implies lower signal thresholds in ADHD exist across all the flash strengths with a stronger influence of the ON-pathway (*p* < .00001) compared to the OFF-pathway (*p* = .0038).

A significantly larger PhNR p72 amplitude was observed in the ADHD than in the control group at the higher flash strengths. In contrast, there were no significant differences in any of the PhNR parameters between the ASD and control groups. This finding had previously been reported [56] and confirms the marker does not clearly discriminate between these clinical conditions. The PhNR parameters are associated with the retinal ganglion cell activity which can be also measured from the central retina using the Pattern ERG. Notwithstanding, neither b-wave time-to-peak or PhNRp72 parameters were as significant as the b-wave amplitude in differentiating ADHD from ASD.

Several studies have examined the signalling interactions between dopamine and glutamate in ADHD [48, 86, 87]. Dopamine acts in the retina as a neurotransmitter for laterally connecting cells, such as horizontal cells and some amacrine cells. Dopaminergic activity in the visual system is reduced in Parkinson’s disease and is characterised by a diminished b-wave amplitude [88–90], implying abnormalities in dopamine signalling could provide an explanation for our findings. In humans, the administration of L-DOPA increases the b-wave amplitude and that observation is consistent with the finding that increasing dopamine levels with methylphenidate reduces retinal noise and improves clinical symptoms in ADHD [46]. We therefore anticipated a relationship might have been found between b-wave amplitude and the administration of systemic methylphenidate. In a small subsample of children with ADHD, we repeated the ERG measures before and after methylphenidate administration and found elevated b-wave amplitudes distinguished ADHD from ASD, regardless of systemic methylphenidate levels. The signalling interaction between dopaminergic and glutamatergic systems for the large b-wave is unlikely to account for the larger b-wave in ADHD.

Measuring the severity of ASD and ADHD from a combination of parental/school reports and observation, we did not find any significant association with either b-wave amplitudes or PhNR parameters. Nor did we find any association between these variables and other ERG measures. ERG parameters were independent of both ASD and ADHD severity as well as measures of intelligence, although there was a stronger relationship between the ERG parameters and the ADHD phenotypes.

### Limitations

The sample of children with ADHD was small, in part because of our stringency to exclude any participant with associated ASD traits. Replication studies with a larger ADHD sample size across a wider age range could identify the age at which ERG abnormalities emerge, and track their development over time, especially in later adolescence and early adulthood. Collecting a complete set of phenotypic data in all three groups will improve the understanding of the relationship between the ERG measures and the phenotypes. The relationship between ADHD symptoms and biological sex is known to be complex, with evidence that in girls, the phenotype is more inattentive in character and thus clinically elusive [91]. A larger sex-matched sample should allow the investigation of the ERG as a potential biomarker in girls for whom the diagnosis of ADHD is uncertain. Our findings did not indicate any impact of stimulant medication on retinal dysfunction, but this observation needs to be replicated with a larger sample because there is potentially an influence of the retina’s dopaminergic network on the b-wave amplitude. Other non-stimulant medications, such as atomoxetine, should also be assessed. Exploring higher flash strengths may reveal greater group differences, but this would require pupil dilation to increase retinal illumination. We deliberately selected ASD and ADHD cohorts in this study that did not have clinically significant traits of both conditions. We also excluded children with significant emotional or behavioural comorbidities. Substantial proportions of clinically identified children with both ASD and ADHD have combined neurodevelopmental disorders as well as other mental health disorders; our protocol should be extended to investigate these conditions in combination too.

## Conclusions

This study was the first to present the observation, from a robust light-adapted ERG measure, that increases in b-wave amplitude can distinguish children with ADHD from those with ASD and typical controls. The observation of a diametrically opposite pattern of b-wave response in the two conditions implies a biomarker exists that correlates with their clinical characteristics. Consequently, the ERG waveform appears to be a physiological marker that could potentially support their differential diagnosis. Whilst the biological significance of the retinal response requires further investigation, our findings imply the differentiation between the clinically related conditions ASD and ADHD could lie in the balance between the activities of glutamatergic and GABAergic neurotransmitter systems.

## Supplementary Information

**Additional file 1: Table S1.** Comorbidities and medications. **Fig. S1.** Iris colour index. **Table S2.** Generalised estimating equations analysis of all the variables. **Table S3.** Pairwise comparisons between groups. **Table S4.** The b-wave amplitude, b-time-to-peak and p72 values. **Fig. S2.** Summary of the ERG measures. **Table S5.** The effects of ADHD medications. **Fig. S3.** The effects of ADHD medications on ERG measures. **Table S6.** Correlations of the ERG measures and phenotypes. **Fig. S4.** Correlation plot network. **Fig. S5.** ROC Curve. **Table S7.** Comparisons of AUC, Cut-off point, Specificity and Sensitivity.

## Data Availability

The datasets, the R codes for the analysis and other analytical outputs of this study are available in the FigShare repository (https://figshare.com/s/5176e951c419612e6273).

## Abbreviations

3Di: The Developmental, Dimensional and Diagnostic Interview
a-amp: a-wave amplitude
ADHD: Attention Deficit Hyperactivity Disorder
ADOS: Autism Diagnostic Observation Schedule
AMPA: α-Amino-3-hydroxy-5-methyl-4-isoxazolepropionic Acid
ASD: Autism Spectrum Disorder
a-time: a-wave time-to-peak
AUC: Area Under the Curve
b-amp: b-wave amplitude
b-time: b-wave time-to-peak
CI: Confidence Interval
CNV: Copy Number Variant
DSM-5: The Diagnostic and Statistical Manual of Mental Disorders, Fifth Edition
DSM-IV: The Diagnostic and Statistical Manual of Mental Disorders, Fourth Edition
ERG: Electroretinogram
GABA: Gamma-aminobutyric Acid
GEE: Generalised Estimating Equations
Glx: Glutamate/glutamine
ICD-10: International Statistical Classification of Diseases and Related Health Problems, Tenth Revision
iGLUR4: Ionotropic Glutamate Receptor 4
IQ: Intelligence Quotient
ISCEV: International Society of Clinical Electrophysiology of Vision
LA: Light-adapted
LA-ERG: Light-adapted Electroretinogram
LA3: ISCEV standard flash 3.0 cd.s.m^−2^
mGLUR: Metabotropic Glutamate Receptor
RETeval: Electroretinogram device from LKC Technologies Inc., USA
PhNR: Photopic Negative Response
ROC: Receiver Operating Characteristic
TRPM1: Transient Receptor Potential cation channel subfamily M member 1
