# Supplementary material for: The electroretinogram b-wave amplitude: a differential physiological measure for Attention Deficit Hyperactivity Disorder and Autism Spectrum Disorder
Source: J Neurodev Disord. 2022 May 6;14:30. doi: 10.1186/s11689-022-09440-2 (PMC9077889; doi:10.1186/s11689-022-09440-2)
Supplement: Supplementary file 1 — Additional file 1: Table S1. Comorbidities and medications. Fig. S1. Iris colour index. Table S2. Generalised estimating equations analysis of all the variables. Table S3. Pairwise comparisons between groups. Table S4. The b-wave amplitude, b-time-to-peak and p72 values. Fig. S2. Summary of the ERG measures. Table S5. The effects of ADHD medications. Fig. S3. The effects of ADHD medications on ERG measures. Table S6. Correlations of the ERG measures and phenotypes. Fig. S4. Correlation plot network. Fig. S5. ROC Curve. Table S7. Comparisons of AUC, Cut-off point, Specificity and Sensitivity. [file 11689_2022_9440_MOESM1_ESM.docx]

**Supplementary Information**

**The electroretinogram b-wave amplitude: a differential physiological measure for Attention Deficit Hyperactivity Disorder and Autism Spectrum Disorder**

Irene O Lee^1^, David H Skuse^1^, Paul A Constable^2^, Fernando Marmolejo-Ramos^3^, Ludvig R Olsen^4^, Dorothy A Thompson^5,6^

1 Behavioural and Brain Sciences Unit, Population Policy and Practice Programme, UCL Great Ormond Street Institute of Child Health, University College London, London, UK.

2 Caring Futures Institute, College of Nursing and Health Sciences, Flinders University, South Australia, Adelaide, Australia.

3 Centre for Change and Complexity in Learning, University of South Australia, Adelaide, Australia.

4 Department of Molecular Medicine (MOMA), Aarhus University, Aarhus, Denmark.

5 The Tony Kriss Visual Electrophysiology Unit, Clinical and Academic Department of Ophthalmology, Sight and Sound Centre, Great Ormond Street Hospital for Children NHS Trust, London, UK.

6 UCL Great Ormond Street Institute of Child Health, University College London, London, UK.

**Corresponding author:** Irene O Lee, [irene.lee@ucl.ac.uk](mailto:irene.lee@ucl.ac.uk)

**Table of Contents**

[**Table S1. Comorbidities and medications** 1](#_Toc101529308)

[**Figure S1. Iris colour index** 3](#_Toc101529309)

[**Table S2. Generalised estimating equations analysis of all the variables** 4](#_Toc101529310)

[**Table S3. Pairwise comparisons between groups** 6](#_Toc101529311)

[**Table S4. The b-wave amplitude, b-time-to-peak and p72 values** 7](#_Toc101529312)

[**Figure S2. Summary of the ERG measures** 9](#_Toc101529313)

[**Table S5. The effects of ADHD medications** 13](#_Toc101529314)

[**Figure S3. The effects of ADHD medications on ERG measures** 14](#_Toc101529315)

[**Table S6. Correlations of the ERG measures and phenotypes** 16](#_Toc101529316)

[**Figure S4. Correlation plot network** 18](#_Toc101529317)

[**Figure S5. ROC Curve** 19](#_Toc101529318)

[**Table S7. Comparisons of AUC, Cut-off point, Specificity and Sensitivity.** 20](#_Toc101529319)

[**Bibliography** 21](#_Toc101529320)

# **Table S1. Comorbidities and medications**

Table S1A. The comorbidities and medications of some participants in ADHD and ASD cohorts.

|  | ADHD | ASD |
| --- | --- | --- |
| Comorbidities | 2 had dyslexia | none had ADHD |
|  | 1 had Tourette | 1 had anorexia |
|  | 1 had Tourette and depression | 1 had Obsessive compulsive disorder (OCD) |
|  | 1 had oppositional defiant disorders | 1 had OCD and dyslexia |
|  | 1 had language disorder and learning difficulties | 1 had myalgic encephalomyelitis |
| Medications | 7 required methylphenidate (3 Concerta XL, 2 Medikinet XL, 2 Equasym XL), 2 took it before testing | 4 had selective serotonin reuptake inhibitors (2 also took methylphenidate and 1 took antihistamines/asthma inhalers) |
|  | 3 required medicine for respiratory conditions (1 took it before testing) | 1 took dopamine antagonist and antihistamines/asthma inhalers |
|  | 1 took insulin before testing | 2 took alpha-2 agonists |
|  |  | 1 had antiepileptic medication |

Table S1B. The ERG testing of ADHD Medication, methylphenidate, in five ADHD participants

| Subject Code | Methylphenidate (MPH) * | ERG Testing after taken MPH | Time elapsed since taken MPH | Day between 2 ERG testing | * Immediate-release : extended-release | Action Duration (hrs) |
| --- | --- | --- | --- | --- | --- | --- |
| AD01 | 54mg Concerta XL | 5hrs | 2 weeks | Different days | 40 : 60 | 12 |
| AD04 | 36mg Concerta XL | 6hrs | 2 days | Different days | 40 : 60 | 12 |
| AD10 | 18mg Concerta XL | 3hrs | 1 month | Same day | 40 : 60 | 12 |
| AD08 | 20mg Medikinet XL | 2hrs | 1 day | Different days | 50 : 50 | 8 |
| AD15 | 60mg Equasym XL | 3hrs | 1 day | Same day | 30 : 70 | 8 |

*Each consists of an immediate-release and an extended-release components which prolong half-life of medication (1).

# **Figure S1. Iris colour index**

Figure S1 shows the distribution of the iris colour index in each group. Both eyes of all participants are included in the counts. There are no significant differences in the iris colour index across the three groups (median differences, *p*=.05).


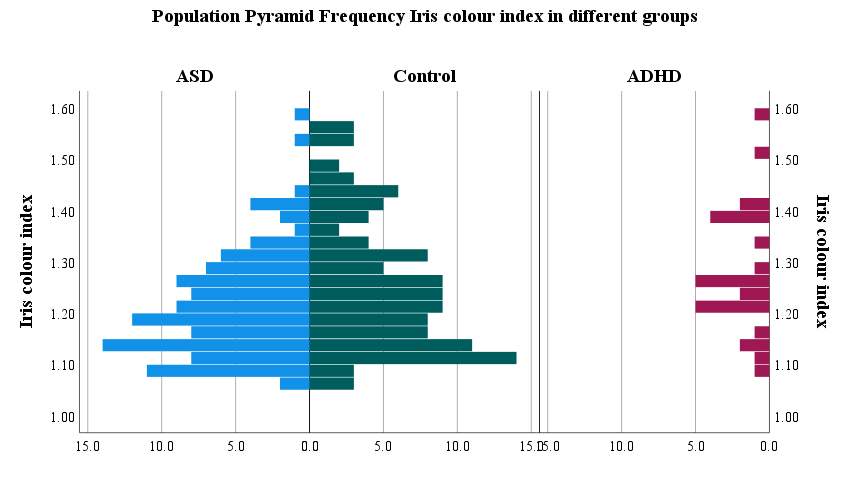


# **Table S2. Generalised estimating equations analysis of all the variables**

Table S2. Table of Wald tests (χ^2^) for sequentially added terms. The *p*-values associated with the Wald statistics are shown in parentheses. The degrees of freedom were *FS* = 9, *V* = 4, *G* = 2, *E* = 4, and *FS•G* = 18; the degrees of freedom was 1 for all other variables. Significant results with * *p*<.005. IV = independent variables. DV = dependent variables. QIC = quasi-likelihood under the independence model information criterion (the lower the better). QIC_2_ = QIC for models in which the interaction *FS•G* was significant and other statistically significant variables were added. % = row-wise percentage of times each variable was significant across the dependent variables and GEE models. **Variable *E* ‘ethnicity’ is excluded owing to imbalance ethnicity distribution (see Table 1). CV = results of the cross-validation analyses on selected models (values are presented in the format ‘RMSE, NRMSE[IQR]’ such that RMSE is the Root Mean Square Error and NRMSE[IQR] is Normalised RMSE [by target IQR]).

| **IV** | **ERG DV** | | | | | | | | | **%** |
| --- | --- | --- | --- | --- | --- | --- | --- | --- | --- | --- |
|  | ***a-time*** | ***a-amp*** | ***b-time*** | ***b-amp*** | ***p72*** | ***Tmin*** | ***PhNRmin*** | ***p.ratio*** | ***w.ratio*** |  |
| ***FS*** | 1082 (2*e*^-16^)***** | 954 (2*e*^-16^)***** | 9285 (2*e*^-16^)***** | 1294 (2*e*^-16^)***** | 465 (2*e*^-16^)***** | 20.85 (.01) | 315.5 (2*e*^-16^)***** | 142.3 (2*e*^-16^)***** | 53.9 (2*e*^-8^)***** | 90 |
| ***V*** | 2 (.80) | 21 (<.001)***** | 4 (.43) | 15 (.004)***** | 10 (.04) | .83 (.93) | 11.7 (.02) | 3.9 (.42) | 4.5 (.34) | 20 |
| ***G*** | 5 (.06) | 14 (.001)***** | 3 (.19) | 24 (5.9*e*^-6^)***** | 4 (.14) | 1.86 (.39) | 6.4 (.04) | 3.9 (.14) | 5.7 (.05) | 30 |
| ***E***** | 25 (6*e*^-5^)***** | 5 (.25) | 51 (2.5*e*^-10^)***** | 14 (.006) | 26 (3.2*e*^-5^)***** | 12.78 (.01) | 14.7 (.0054) | 20.4 (.004)***** | 18.7 (<.001)***** | 50 |
| ***I*** | 1 (.40) | 3 (.09) | 0 (.76) | 0 (.85) | 3 (.09) | .08 (.77) | 2.4 (.11) | .8 (.36) | 3.3 (.07) | 0 |
| ***s*** | 1 (.32) | 0 (.55) | 1 (.26) | 4 (.04) | 1 (.37) | 9.56 (.002)***** | .3 (.60) | 1.8 (.17) | 0 (.83) | 10 |
| ***M*** | 2 (.18) | 2 (.11) | 0 (.90) | 2 (.19) | 7 (.009) | .31 (.57) | 8.2 (.004)***** | 6.3 (.01) | 10.7 (.001)***** | 20 |
| ***A*** | 1 (.24) | 2 (.21) | 6 (.01) | 0 (.61) | 0 (.65) | .72 (.39) | 1.4 (.22) | .5 (.49) | .3 (.56) | 0 |
| ***e*** | 2 (.16) | 0 (.79) | 11 (<.001)***** | 0 (.97) | 0 (.66) | 4.17 (.04) | .4 (.54) | 0 (.97) | .8 (.38) | 10 |
| ***FS•G*** | 27 (.07) | 23 (.17) | 73 (1.4*e*^-8^)* | 63 (7.8*e*^-7^)***** | 41 (.001)***** | 30.31 (.03) | 32.5 (.01) | 34.7 (.01) | 31.4 (.02) | 30 |
|  |  |  |  |  |  |  |  |  |  |  |
| **QIC** | 3894 | 3937 | 4061 | 3956 | 4027 | 4008 | 4017 | 3888 | 3934 |  |
| **QIC_2_** |  |  | 3941 | 3863 | 3973 |  |  |  |  |  |
| **CV** |  |  | 3.10 (.60) *_G_* | 11.3 (.69) *_G_* | 4.69 (.87) *_G_* |  |  |  |  |  |
|  |  |  | 1.14 (.22) *_FS_* | 9.41 (.58) *_FS_* | 4.43 (.82) *_FS_* |  |  |  |  |  |
|  |  |  | 1.14 (.22) *_FS•G_* | 8.84 (.54) *_FS•G_* | 4.42 (.82) *_FS•G_* |  |  |  |  |  |
|  |  |  | 1.14 (.22*) _FS+G_* | 8.84 (.54) *_FS+G_* | 4.40 (.82) *_FS+G_* |  |  |  |  |  |

# **Table S3. Pairwise comparisons between groups**

Table S3. Results of nonparametric multiple comparisons between groups at each flash strength in each of the dependent variables (DV). * Significant pairwise comparisons at *p*<.005. C = Control; a = ASD; A = ADHD. *s (p)* = statistic of the nonparametric multiple comparisons test (associated *p*-value). *op* = overall *p*-value.

| **Flash strength** | **Contrast** | **DV** | | | | | |
| --- | --- | --- | --- | --- | --- | --- | --- |
|  |  | ***b-amp*** | | ***b-time*** | | ***p72*** | |
|  |  | *s (p)* | *op* | *s (p)* | *op* | *s (p)* | *op* |
| -.367 | C - a | 1.96 (.12) | 6.21*e*^-5^* | -1.91 (.13) | .010 | -.75 (.72) | .160 |
|  | A - a | 4.63 (6.21*e*^-5^)* |  | 1.48 (.30) |  | -1.83 (.16) |  |
|  | A - C | 3.45 (2.49*e*^-3^)* |  | 2.88 (.01) |  | -1.34 (.37) |  |
| -.119 | C - a | 1.15 (.48) | 1.77*e*^-7^* | -2.31 (.058) | .002* | -0.74 (.73) | 6.04*e*^-5^* |
|  | A - a | 5.97 (1.77*e*^-7^)* |  | 1.80 (.17) |  | -4.53 (6.04*e*^-5^)* |  |
|  | A - C | 5.33 (6.62*e*^-6^)* |  | 3.42 (.002) |  | -4.12 (2.43*e*^-4^)* |  |
| .114 | C - a | 1.46 (.31) | 7.37*e*^-8^* | -2.65 (.02) | .009 | 0.27 (.95) | .019 |
|  | A - a | 6.14 (7.37*e*^-8^)* |  | 1.10 (.51) |  | -2.53 (.03) |  |
|  | A - C | 5.33 (2.37*e*^-6^)* |  | 3.01 (.009) |  | -2.73 (.01) |  |
| .398 | C - a | 2.26 (6.53*e*^-2^)* | 4.87*e*^-11^* | -2.93 (.01) | .011 | -0.06 (.99) | .0011* |
|  | A - a | 7.70 (4.87*e*^-11^)* |  | -1.90 (.14) |  | -3.62 (.001)* |  |
|  | A - C | 6.11 (5.32*e*^-8^)* |  | .20 (.97) |  | -3.67 (.001)* |  |
| .477 | C - a | 1.22 (.44) | 2.48*e*^-13^* | -1.50 (.29) | .170 | 0.21 (.97) | <.001* |
|  | A - a | 8.35 (2.48*e*^-13^)* |  | -1.79 (.17) |  | -3.45 (.002)* |  |
|  | A - C | 7.57 (3.19*e*^-11^)* |  | -.72 (.75) |  | -3.71 (<.001)* |  |
| .602 | C - a | 2.15 (.08) | 3.33*e*^-16^* | -2.49 (.03) | .037 | -0.29 (.95) | .040 |
|  | A - a | 9.38 (3.33*e*^-16^)* |  | -2.3 (.058) |  | -2.44 (.04) |  |
|  | A - C | 7.66 (8.31*e*^-12^)* |  | -.48 (.87) |  | -2.28 (.06) |  |
| .799 | C - a | 1.78 (.17) | 7.99*e*^-15^* | -2.83 (.015) | .003* | -0.79 (.70) | .690 |
|  | A - a | 8.99 (7.99*e*^-15^)* |  | -3.85 (.003)* |  | -0.81 (.69) |  |
|  | A - C | 7.37 (3.52*e*^-11^)* |  | -1.32 (.38) |  | -0.28 (.95) |  |
| .949 | C - a | 1.39 (.35) | 1.88*e*^-13^* | -2.29 (.06) | .024 | -1.60 (.24) | .008 |
|  | A - a | 8.34 (1.88*e*^-13^)* |  | -2.66 (.02) |  | -3.06 (.008) |  |
|  | A - C | 7.30 (1.24*e*^-10^)* |  | -1.05 (.54) |  | -2.00 (.11) |  |
| 1.114 | C - a | 1.90 (.14) | 2.32*e*^-7^* | -2.25 (.06) | .066 | .23 (.96) | 6.21*e*^-5^* |
|  | A - a | 5.79 (2.32*e*^-7^)* |  | -1.23 (.43) |  | -4.32 (1.11*e*^-4^)* |  |
|  | A - C | 4.49 (5.40*e*^-5^)* |  | .46 (.88) |  | -4.57 (6.21*e*^-5^)* |  |
| 1.204 | C - a | 3.17 (5.97*e*^-3^)* | 3.98*e*^-12^* | -2.94 (.01) | .001* | -0.77 (.71) | .003* |
|  | A - a | 8.11 (3.98*e*^-12^)* |  | -3.60 (.001)* |  | -3.36 (.003)* |  |
|  | A - C | 5.76 (6.03*e*^-7^)* |  | -1.60 (.24) |  | -2.86 (.01) |  |

# **Table S4. The b-wave amplitude, b-time-to-peak and p72 values**

Table S4A shows the mean±SD (Mdn) of the b-wave amplitudes (µV) in each group at each flash strength. * *p*<.005, statistically significant difference amongst the three groups.

| Flash Strength (log phot cd.s.m^-2^) | ADHD | Control | ASD | Overall *p*-value |
| --- | --- | --- | --- | --- |
| -.367 | 14.59±5.2 (14.8) | 11.98±3.8 (11.2) | 11.07±4.2 (10.4) | *6.21e^-5^** |
| -.119 | 21.59±7.3 (21.5) | 15.95±4.3 (15.6) | 15.24±5.6 (14.6) | *1.77e^-7^** |
| .114 | 33.99±11.4 (34.9) | 25.59±7.1 (25.1) | 24.13±9.0 (23.5) | *7.37e^-8^** |
| .398 | 43.62±11.9 (45.0) | 33.04±9.1 (31.6) | 30.14±9.6 (29.6) | *4.87e^-11^** |
| .477 | 44.16±15.1 (42.0) | 30.16±9.7 (29.1) | 28.14±10.2 (27.6) | *2.48e^-13^** |
| .602 | 44.34±12.8 (40.1) | 32.86±9.5 (32.1) | 30.06±10.1 (28.9) | *3.33e^-16^** |
| .799 | 40.63±11.6 (39.1) | 30.14±9.3 (29.0) | 27.87±9.2 (27.2) | *7.99e^-15^** |
| .949 | 39.44±10.0 (37.8) | 29.45±8.4 (29.2) | 27.49±9.4 (28.2) | *1.88e^-13^** |
| 1.114 | 36.36±10.9 (34.4) | 29.12±8.0 (28.8) | 26.85±9.4 (27.0) | *2.32e^-7^** |
| 1.204 | 37.20±10.3 (36.9) | 28.22±8.6 (28.6) | 24.66±8.9 (24.2) | *3.98e^-12^** |

Table S4B. The mean±SD (Mdn) of the b-wave time-to-peak (ms) in each group at all flash strengths. * *p*<.005, statistically significant difference amongst the three groups.

| Flash Strength (log phot cd.s.m^-2^) | ADHD | Control | ASD | Overall *p*-value |
| --- | --- | --- | --- | --- |
| -.367 | 22.33±1.0 (22.1) | 21.94±1.2 (21.9) | 22.31±2.2 (22.0) | .010 |
| -.119 | 23.62±1.1 (23.7) | 23.15±1.0 (23.2) | 23.48±1.3 (23.5) | *.002** |
| .114 | 24.87±.9 (24.6) | 24.48±.6 (24.4) | 24.80±.9 (24.4) | .009 |
| .398 | 25.81±.8 (26.0) | 25.88±.8 (26.0) | 26.24±1.0 (26.2) | .011 |
| .477 | 28.06±1.0 (28.0) | 28.20±.9 (28.1) | 28.4±1.2 (28.2) | .170 |
| .602 | 27.18±.7 (26.9) | 27.26±.8 (27.3) | 27.6±1.0 (27.7) | .037 |
| .799 | 28.4±.9 (28.3) | 28.60±.9 (28.4) | 28.0±1.1 (28.7) | *.003** |
| .949 | 29.24±1.0 (29.1) | 29.41±1.1 (29.4) | 29.74±1.1 (29.8) | .024 |
| 1.114 | 30.30±1.0 (30.2) | 30.23±1.1 (30.2) | 30.54±1.5 (30.3) | .066 |
| 1.204 | 30.64±1.1 (30.3) | 30.79±1.1 (30.7) | 31.16±1.8 (31.2) | *.001** |

Table S4C. The mean±SD (Mdn) of the Photopic negative response p72 amplitude (µV) in each group at all flash strengths. * *p*<.005, statistically significant difference amongst the three groups.

| Flash Strength (log phot cd.s.m^-2^) | ADHD | Control | ASD | Overall *p*-value |
| --- | --- | --- | --- | --- |
| -.367 | -3.43±4.8 (-3.0) | -2.92±4.2 (-2.3) | -2.61±4.2 (-2.2) | .160 |
| -.119 | -6.06±4.3 (-5.3) | -3.76±3.0 (-3.4) | -3.54±4.3 (-2.9) | *6.04e^-5^** |
| .114 | -6.02±5.6 (-6.1) | -4.01±3.66 (-3.6) | -4.41±3.6 (-4.5) | .019 |
| .398 | -8.94±9.9 (-6.4) | -4.92±4.0 (-4.8) | -5.21±4.5 (-4.7) | *.001** |
| .477 | -8.28±5.7 (-7.4) | -5.97±4.5 (-5.2) | -6.01±5.0 (-5.8) | *<.001** |
| .602 | -7.84±4.6 (-7.5) | -6.04±4.7 (-5.6) | -5.7±3.9 (-5.5) | .040 |
| .799 | -7.28±5.0 (-6.8) | -7.00±4.2 (-6.8) | -6.77±4.1 (-6.3) | .690 |
| .949 | -8.34±5.5 (-8.5) | -7.35±3.9 (-3.4) | -6.59±4.5 (-6.4) | .008 |
| 1.114 | -10.57±6.3 (-10.2) | -7.27±3.6 (-7.1) | -7.45±4.5 (-7.2) | *6.21e^-5^** |
| 1.204 | -9.39±4.8 (-9.7) | -7.96±4.2 (-7.4) | -7.40±4.5 (-7.2) | *.003** |

# **Figure S2. Summary of the ERG measures**

Boxplots of each ERG parameter against all the flash strengths of the three groups are presented in each figure subset. The median lines and mean triangles with range within 1.5 interquartile range are plotted in each boxplot. There are no significant differences among the three groups in these ERG parameters. CTL = Control.

Figure S2A.


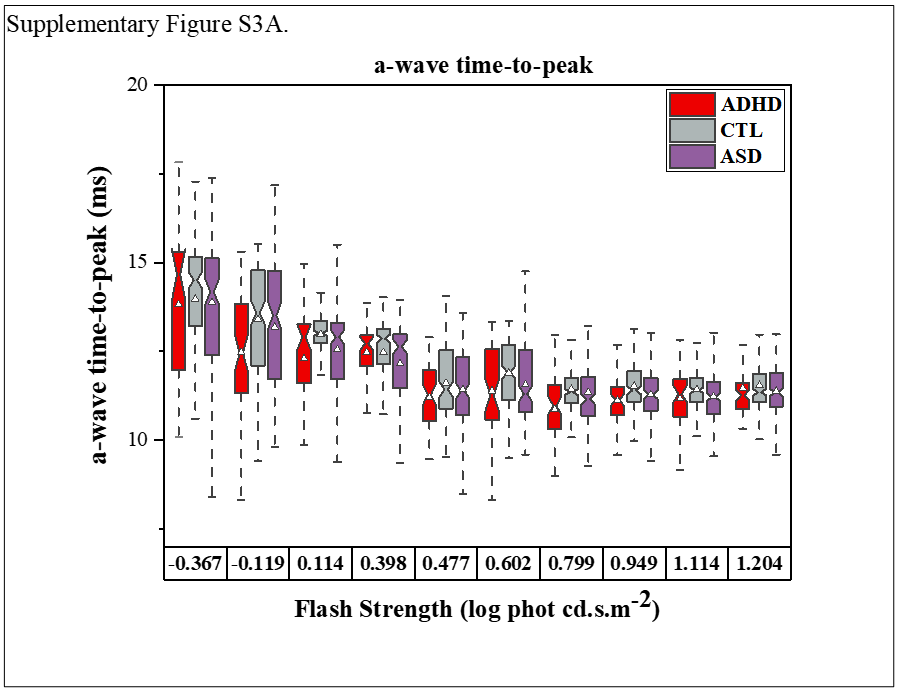


Figure S2B


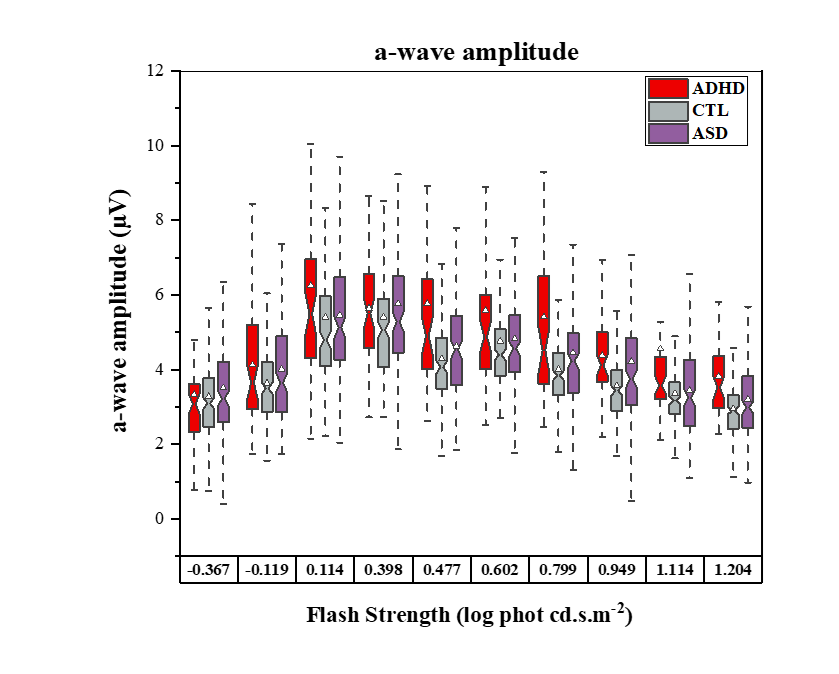


Figure S2C.


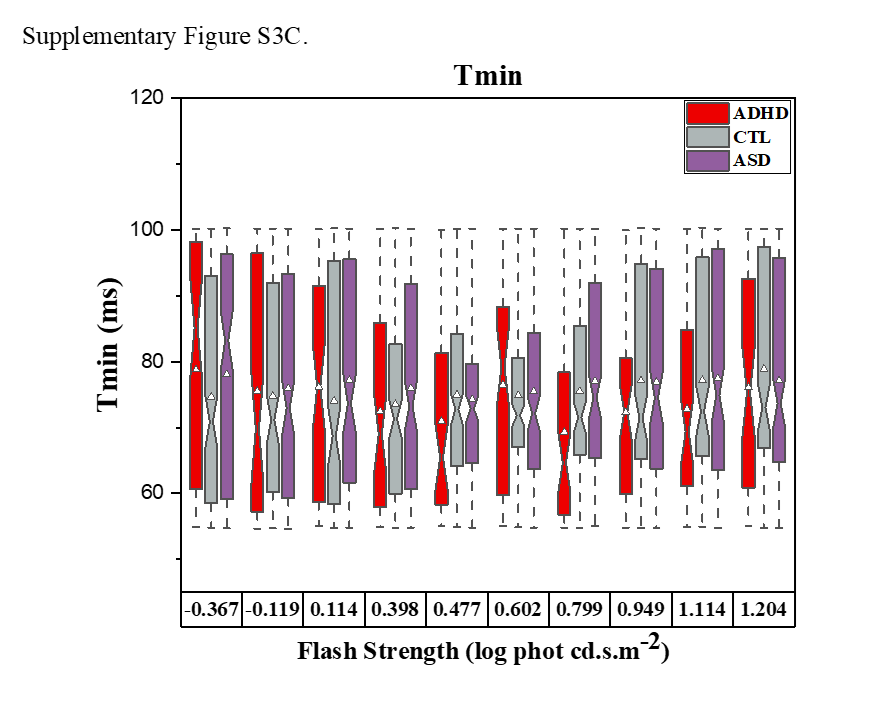


Figure S2D.


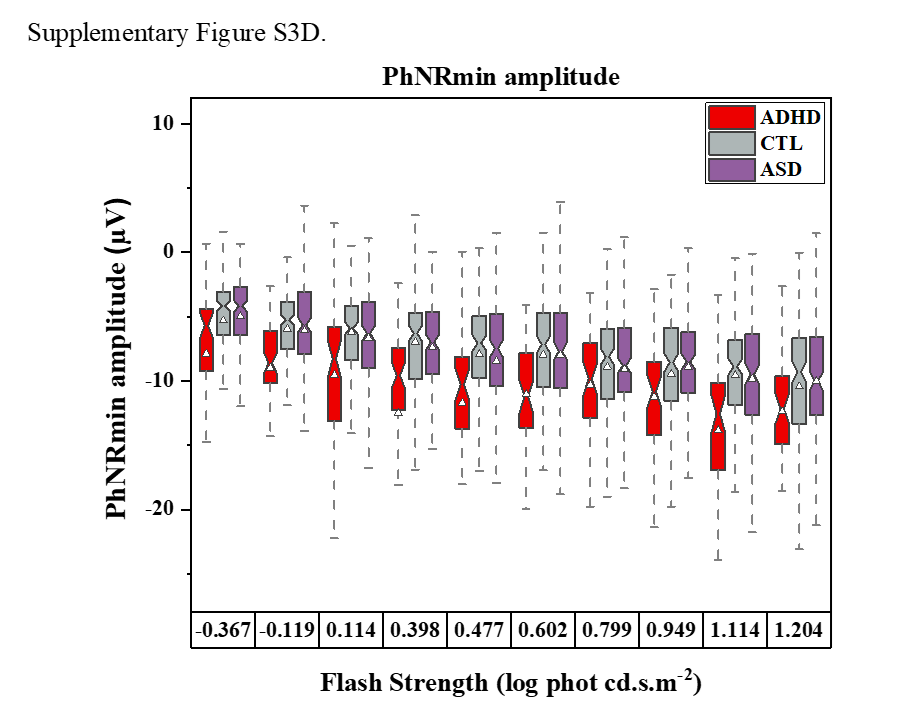


Figure S2E.


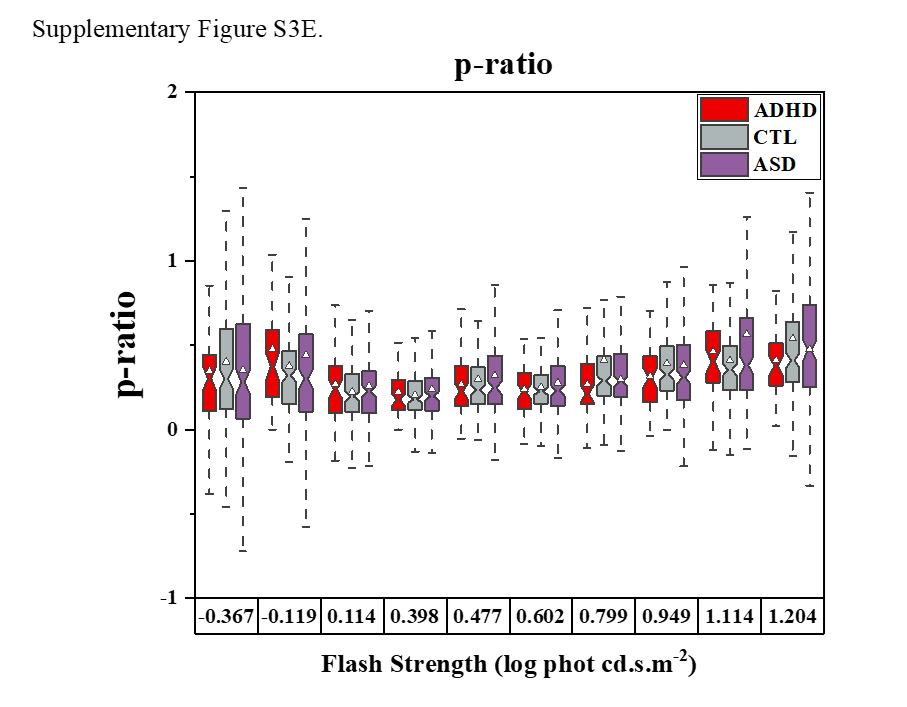


Figure S2F.


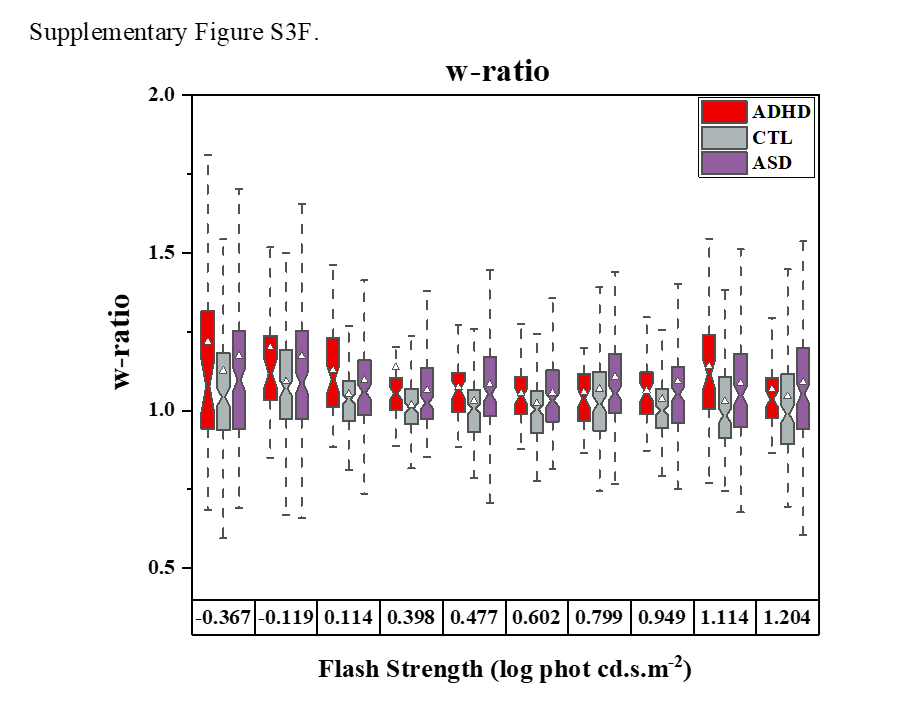


# **Table S5. The effects of ADHD medications**

Table S5. The effects of taking ADHD medications before and after the ERG testing. Results of the Wald chi-squared (χ^2^) tests (ANOVA Type 3) of the robust linear models. The *p*-values associated to the chi-squared statistics are shown in parenthesis. The degrees of freedom were *FS* = 9, *V* and *E* = 2, and *FS•G* = 9; the degrees of freedom was 1 for all other variables. IV = independent variables (*M_ba_* = medication before and after. Other labels as in the main document). DV = dependent variables (labels as in the main document). Significant results with * *p*<.005. The finding of interest is no significant main effect of *M_ba_* and no significant interaction between flash strength and *M_ba_*.

| **IV** | **DV** | | | | | | | | |
| --- | --- | --- | --- | --- | --- | --- | --- | --- | --- |
|  | ***a-time*** | ***a-amp*** | ***b-time*** | ***b-amp*** | ***p72*** | ***Tmin*** | ***PhNRmin*** | ***p.ratio*** | ***w.ratio*** |
| *M_ba_* | .05 (.82) | .14 (.70) | 1.73 (.18) | .79 (.37) | 1.73 (.18) | 8.40 (.003) * | 1.81 (.17) | 1.36 (.24) | .06 (.79) |
| *FS* | 136.0 (<.001)* | 170.9 (<.001)* | 4876.2 (<.001)* | 670.0 (<.001)* | 77.31 (<.001)* | 16.16 (.063) | 52.66 (<.001)* | 45.03 (<.001)* | 20.50 (.015) |
| *V* | 12.58 (.001)* | 10.08 (.006) | 1.65 (.43) | 14.65 (<.001)* | 2.50 (.28) | .32 (.84) | 3.16 (.20) | .76 (.68) | 7.18 (.027) |
| *E* | 4.47 (.10) | 12.80 (.001)* | 93.7 (<.001)* | 112.5 (<.001)* | 5.94 (.051) | 115.78 (<.001)* | 10.01 (.006) | .89 (.64) | 4.03 (.13) |
| *I* | .92 (.33) | .82 (.36) | .01 (.91) | .00 (.99) | 5.53 (.01) | .71 (.39) | 6.63 (.009) | 1.55 (.21) | 1.69 (.19) |
| *s* | .03 (.84) | 23.04 (<.001)* | 5.99 (.014) | 156.4 (<.001)* | 5.37 (.02) | 8.26 (.004)* | 3.46 (.062) | .67 (.41) | 1.40 (.23) |
| *A* | .10 (.74) | 7.83 (.0051) | 5.37 (.020) | 87.02 (<.001)* | 3.81 (.050) | 4.56 (.032) | 2.14 (.14) | .09 (.75) | .02 (.88) |
| *e* | 3.52 (.060) | 1.47 (.22) | 2.51 (.11) | 7.36 (.006) | 1.65 (.19) | .00 (.98) | 3.72 (.053) | .20 (.64) | 3.79 (.051) |
| *FS•M_ba_* | 2.88 (.96) | 5.86 (.75) | 5.69 (.76) | 12.94 (.16) | 9.85 (.36) | 9.80 (.36) | 12.14 (.20) | 10.62 (.30) | 21.27 (.011) |

# **Figure S3. The effects of ADHD medications on ERG measures**

Figure S3. A summary of all ERG measures before and after taken ADHD medications (methylphenidate) in five ADHD participants. Mean and standard error bars of the repeat measures of both eyes are plotted at each flash strength. There are no statistically significant differences of all the ERG parameters between before and after taken methylphenidate.

# **Table S6. Correlations of the ERG measures and phenotypes**

Table S6 shows the correlations of the ADHD and ASD phenotypes with the ERG measures at 1.204 log phot cd.s.m^-2^. All the repeat measures of both eyes in each participant were averaged before the correlation analysis.

| **At 1.204 log phot cd.s.m^-2^** | | ***a-time*** | ***a-amp*** | ***b-time*** | ***b-amp*** | ***p72*** | ***Tmin*** | ***PhNRmin*** | ***p-ratio*** | ***w-ratio*** |
| --- | --- | --- | --- | --- | --- | --- | --- | --- | --- | --- |
| **Hyperactivity** | Pearson Correlation | 0.210 | -0.071 | 0.014 | 0.286 | -0.151 | 0.299 | -0.116 | 0.041 | -0.011 |
|  | Sig. (2-tailed) | 0.241 | 0.694 | 0.941 | 0.106 | 0.402 | 0.090 | 0.519 | 0.819 | 0.953 |
|  | N | 33 | 33 | 33 | 33 | 33 | 33 | 33 | 33 | 33 |
| **Impulsivity** | Pearson Correlation | 0.37 | 0.405 | -0.098 | -0.166 | -0.106 | 0.192 | -0.042 | 0.080 | 0.477 |
|  | Sig. (2-tailed) | 0.034 | 0.020 | 0.589 | 0.356 | 0.558 | 0.286 | 0.816 | 0.656 | *0.005* |
|  | N | 33 | 33 | 33 | 33 | 33 | 33 | 33 | 33 | 33 |
| **Inattention** | Pearson Correlation | 0.270 | -0.127 | -0.141 | 0.203 | -0.344 | 0.177 | -0.223 | 0.103 | 0.077 |
|  | Sig. (2-tailed) | 0.129 | 0.481 | 0.435 | 0.257 | 0.050 | 0.323 | 0.213 | 0.570 | 0.669 |
|  | N | 33 | 33 | 33 | 33 | 33 | 33 | 33 | 33 | 33 |
| **ADHD Severity** | Pearson Correlation | 0.336 | -0.185 | -0.119 | 0.323 | -0.337 | 0.145 | -0.302 | 0.146 | 0.116 |
|  | Sig. (2-tailed) | 0.045 | 0.281 | 0.491 | 0.055 | 0.044 | 0.398 | 0.073 | 0.394 | 0.499 |
|  | N | 36 | 36 | 36 | 36 | 36 | 36 | 36 | 36 | 36 |
| **ASD_Severity** | Pearson Correlation | -0.103 | 0.159 | -0.163 | -0.156 | -0.155 | -0.346 | -0.171 | 0.266 | 0.339 |
|  | Sig. (2-tailed) | 0.563 | 0.369 | 0.357 | 0.378 | 0.382 | 0.045 | 0.333 | 0.128 | 0.050 |
|  | N | 34 | 34 | 34 | 34 | 34 | 34 | 34 | 34 | 34 |
| **ADOS** | Pearson Correlation | -0.117 | 0.101 | -0.261 | -0.098 | -0.102 | -0.222 | -0.188 | 0.226 | 0.286 |
|  | Sig. (2-tailed) | 0.511 | 0.570 | 0.136 | 0.582 | 0.566 | 0.207 | 0.288 | 0.198 | 0.102 |
|  | N | 34 | 34 | 34 | 34 | 34 | 34 | 34 | 34 | 34 |
| **FSIQ** | Pearson Correlation | 0.181 | -0.105 | 0.285 | 0.009 | 0.017 | -0.076 | 0.037 | 0.057 | -0.061 |
|  | Sig. (2-tailed) | 0.224 | 0.480 | 0.053 | 0.953 | 0.909 | 0.612 | 0.804 | 0.705 | 0.686 |
|  | N | 47 | 47 | 47 | 47 | 47 | 47 | 47 | 47 | 47 |

Note: ADHD Severity = ADHD severity score; ASD severity = autism severity score; ADOS = Autism Diagnostic Observation Schedule total score; FSIQ = Full Scale IQ; Sig. = statistically significant *p<.005*; N = number of individuals (Some participants recruited in the local clinics did not have the phenotypic data).

# **Figure S4. Correlation plot network**

Figure S4 displays the correlation plot network of the ERG parameters and ASD or ADHD phenotypes. The colour mapping vertical bar shows the correlation coefficients. The ERG measures at 1.204 log phot cd.s.m^-2^ were used and their repeat measures of both eyes were averaged before the correlation analysis. A cluster of several ERG parameters are directly linked to the ADHD phenotypes.

# **Figure S5. ROC Curve**

Figure S5. Receiver Operating Characteristic (ROC) curve with sensitivity and specificity of the b-wave amplitude at 0.398 and 1.204 log phot cd.s.m^-2^ to differentiate ADHD from the Control and ASD groups.

**ROC curves of the b-wave amplitude to distinguish ADHD from ASD**


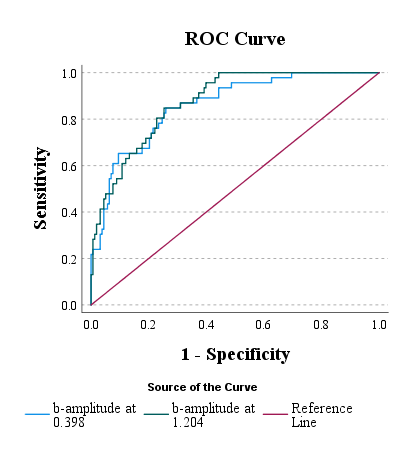


# **Table S7. Comparisons of AUC, Cut-off point, Specificity and Sensitivity.**

Table S7. Comparisons of the Area Under the Curve (AUC), specificity, sensitivity and the cut-off point of the b-wave amplitude at 0.398 and 1.204 log phot cd.s.m^-2^ for distinguishing ADHD from the Control and ASD groups.

| **Flash Strength** |  | **ADHD vs ASD+CTL combined** | **ADHD vs CTL** | **ADHD vs ASD** |
| --- | --- | --- | --- | --- |
| 0.398 log phot cd.s.m^-2^ | AUC /b-amp cut-off point (µV) | 0.84* / 36.4 | 0.81* / 36.4 | 0.86* / 36.2 |
|  | Sensitivity / Specificity | 80% / 71% | 81% / 67% | 80% / 67% |
| 1.204 log phot cd.s.m^-2^ | AUC /b-amp cut-off point (µV) | 0.84* / 30.5 | 0.82* / 33.7 | 0.88* / 30.5 |
|  | Sensitivity / Specificity | 81% / 70% | 80% / 63% | 84% / 57% |

*Asymptotic Significance *p*<0.0001, Null hypothesis: true area=0.5**.**

Note: ASD+CTL combined means combining the ASD and control groups together.

# **Bibliography**

1. Coghill D, Banaschewski T, Zuddas A, Pelaz A, Gagliano A, Doepfner M. (2013): Long-acting methylphenidate formulations in the treatment of attention-deficit/hyperactivity disorder: a systematic review of head-to-head studies. *BMC Psychiatry* 13: 237.
